# DDX17 is required for efficient DSB repair at DNA:RNA hybrid deficient loci

**DOI:** 10.1093/nar/gkac843

**Published:** 2022-10-06

**Authors:** Aldo S Bader, Janna Luessing, Ben R Hawley, George L Skalka, Wei-Ting Lu, Noel F Lowndes, Martin Bushell

**Affiliations:** Cancer Research UK Beatson Institute, Glasgow G61 1BD, UK; Centre for Chromosome Biology, Biomedical Sciences Biulding (BSB), School of Biological & Checmical Sciences, University of Galway, Galway, H91W2TY, Ireland; Department of Pharmacology, Weill Cornell Medicine, Cornell University, New York, NY 10065, USA; Cancer Research UK Beatson Institute, Glasgow G61 1BD, UK; The Francis Crick Institute, London NW1 1AT, UK; Centre for Chromosome Biology, Biomedical Sciences Biulding (BSB), School of Biological & Checmical Sciences, University of Galway, Galway, H91W2TY, Ireland; Cancer Research UK Beatson Institute, Glasgow G61 1BD, UK; Institute of Cancer Sciences, University of Glasgow, Glasgow G61 1QH, UK

## Abstract

Proteins with RNA-binding activity are increasingly being implicated in DNA damage responses (DDR). Additionally, DNA:RNA-hybrids are rapidly generated around DNA double-strand breaks (DSBs), and are essential for effective repair. Here, using a meta-analysis of proteomic data, we identify novel DNA repair proteins and characterise a novel role for DDX17 in DNA repair. We found DDX17 to be required for both cell survival and DNA repair in response to numerous agents that induce DSBs. Analysis of DSB repair factor recruitment to damage sites suggested a role for DDX17 early in the DSB ubiquitin cascade. Genome-wide mapping of R-loops revealed that while DDX17 promotes the formation of DNA:RNA-hybrids around DSB sites, this role is specific to loci that have low levels of pre-existing hybrids. We propose that DDX17 facilitates DSB repair at loci that are inefficient at forming DNA:RNA-hybrids by catalysing the formation of DSB-induced hybrids, thereby allowing propagation of the damage response.

## INTRODUCTION

Accurate repair of DNA damage prevents the mutations that are the driving force behind carcinogenesis. To maintain fidelity our cells have evolved a network of repair pathways that detect and resolve the various forms of DNA damage including: nucleotide adducts, inter-strand crosslinks, collapsed replication forks and strand breaks (1). Double-strand breaks (DSBs) are considered the most toxic form of DNA damage due to their relatively high propensity for causing mutations (2) and, mutations in multiple DSB repair genes are associated with an increased cancer risk (3,4).

The complex signal transduction in response to DSBs involves three DDR kinases: ataxia telangiectasia mutated (ATM), ataxia telangiectasia and rad3-related (ATR) and DNA-dependent protein kinase (DNA-PK) (5,6). An early and essentially universal marker of DSBs is the phosphorylation of Serine 129 of histone H2AX to form γ-H2AX which directly recruits phosphorylated MDC1 to DNA breaks. The E3-ubiquitin ligase RNF8 then binds to MDC1 and mono-ubiquitylates other factors, including L3MBTL2 (7) and possibly the H1 linker histone, thereby creating a marker that recruits the E3 ligase RNF168 (8,9). RNF168 is currently thought to poly-ubiquitylate H1, contributing to decompaction of chromatin, and to mono-ubiquitylate H2A and H2AX at two positions; K13 and K15 (9,10). These H2A ubiquitylations facilitate the recruitment of 53BP1 and BRCA1 that determine pathway choice, either non-homologous end-joining (NHEJ) or homologous recombination (HR), respectively. HR is a high-fidelity replication-based process, whereas NHEJ involves rapid processing and ligation of the broken DNA ends that can be error prone. There is growing evidence that the DDR signalling cascade is more complex as a number of additional factors have been implicated, including additional ubiquitin ligases, such as HUWE1 (11), as well as RNA binding proteins, such as DROSHA and WRAP53β (12,13). Chromatin is not only a barrier to DNA repair but also provides the substrate for post-translational modifications and chromatin remodelling enzymes required for ordered recruitment of both repair factors and the chromatin remodelling enzymes essential for repair of and recovery from DNA damage.

There is emerging evidence for RNA and RNA binding proteins functioning in the DSB response, including DSB repair *per se* (14–16). A wide variety of RNA binding proteins have already been characterised as DNA repair genes including the splicing factor THRAP3 (17) and the DEAD-box helicase Senataxin (18). In addition, both on-going transcription and RNA itself have been implicated in the damage response, with transcription inhibitors (19–21) and RNase treatments (19,22) both being shown to significantly impair the damage response. While DNA:RNA-hybrids have been detected around active DSBs and shown to be required for efficient repair (13,18,20,23), more recently, RNA polymerase III has been shown to be recruited to DSBs where it catalyses transcription templated from the 3′ ssDNA overhang produced upon resection (16). In addition, many RNA binding proteins have been identified that promote their formation and resolution around break sites (13,18,24,25). Defining the roles of RNA and RNA binding proteins in the DDR is an emerging area requiring characterisation.

DDX17 is another DEAD-box helicase known to exist as two isoforms known, p72 and p82, that result from an alternative translation start site. DDX17 has RNA helicase, annealing and branch migration activity allowing it to remodel complex RNA structures (26–30) and is known to function in microRNA biogenesis (26,28,31,32), ribosomal biogenesis (27,28,31) and splicing (27,28). DDX17 interacts with the miRNA processors DROSHA and DGCR8 and remodels the 3′ flanking regions of pri-miRNAs to enhance miRNA processing. In mice, *Ddx17^−/−^* knockout results in early embryonic lethality due to impaired miRNA and rRNA processing (33). Interestingly, a recent report has shown a possible role for DDX17 in resolving or preventing FUS-mediated DNA damage in neuronal cells (34), highlighting potential roles of DDX17 beyond canonical RNA biogenesis functions.

Here, we use a meta-analysis of proteomic screens to identify novel DNA repair proteins and highlight DDX17 as a potential repair factor. Our investigations provide evidence that DDX17 is important for maintaining genome stability via DSB repair, specifically via facilitating RNF168 recruitment and subsequent histone ubiquitylation at DSBs. Consistent with these observations, cells deficient for DDX17 function display impaired DSB repair in both NHEJ and HR pathways resulting in DNA damage accumulating to toxic levels. Additionally, we characterised a requirement for DDX17 in promoting DSB-induced DNA:RNA-hybrid formation. Interestingly, this role is preferentially required around DNA breaks in loci that are naturally deficient for DNA:RNA-hybrids. Our data suggest that DDX17 specifically facilitates DSB repair by promoting DSB-induced hybrid formation at regions of the genome which are inefficient at forming hybrids.

## MATERIALS AND METHODS

### Cell culture

U2OS, A549, DIvA, HeLA and BJ-5ta cells were cultured in Dulbecco's modified Eagle's medium (DMEM, GibCo) supplemented with 10% Fetal bovine serum and 2 mM l-glutamine with DIvA cells also containing 1 μg/ml puromycin. All cells were incubated at 37°C with a 5% CO_2_, humidified atmosphere. hTERT immortalized RPE-1 (ATCC) cells were cultured in DMEM-F12 media supplemented with 10% FBS (Gibco) and 1% PenStep. hTERT-RPE1 cells were verified by STR analysis (Eurofins Genomics). U2OS, U2OS-derived (DIvA, EJ5, HR-U2OS) and hTERT-RPE1 cell lines are female, while A549 and BJ-5ta cell lines are male.

### Transfection and drug treatments

For DIvA, A549 and BJ-5ta cells, Dharmafect 1 was used at a final dilution of 1/1000 and siRNA targeting either DDX17 (Thermo, s2062 for A549 cells, s20623 for all other experiments), RAD54 () or an untargeted control (Horizon Discovery, D-001810-01-05) were used at a final dilution of 20 nM. Cells were cultured for 24 h prior to transfection and treated 48 h after transfection. siRNA and Dharmafect 1 (Horizon Discovery, T-2001-03) were first diluted separately in Optimem to a volume of 5% the final desired medium volume, and incubated for 5min at RT. siRNA and Dharmafect 1 were then combined in 1:1 ratio and incubated for 20 min at RT. The remaining 90% of the final volume of medium was then added and the medium on the cells was immediately replaced with this. For siRNA transfections in hTERT-RPE1, U2OS, HR-U2OS and HeLa cells were transfected with Oligofectamine Reagent (LifeTechnologies) according to manufacturer's instructions. Briefly, 1.2–1.5 × 10^5^ cells were plated on a 35 mm cell culture dish. After 24 h cells were transfected with 40pmol of negative control siRNA (Dharmacon) or siRNA targeting the gene of interest. Cells were treated and harvested 48h post transfection. ATMi (KU55933, SelleckChem) and DNA-PKi (NU7026) were used at 10 uM unless otherwise stated. Olaparib (AZD2281, SelleckChem) and ICRF-193 (I4659, Sigma) were used at indicated doses. Irradiation was performed using a Mainance Millenium Sample Irradiator containing a Cs-137 sealed source.

### Western blotting

Cells were harvested by lysing in 1.2× sample loading buffer and sonicated with a Diagenode Biorupter for 5 min on high to shear the DNA. Samples were run on either 6% or 10% poly-acrylamide gels and transferred in tris-glycine transfer buffer containing 20% methanol and 0.01% SDS onto nitrocellulose membranes. Membranes were blocked using 5% BSA in TBST and primary antibody probing was done overnight at 4°C with 5% BSA in TBST. Antibodies and dilutions are listed in Supplementary Table S1. Membranes were then washed for 10 min in TBST 3 times at RT and incubated with secondary antibodies (Li-COR Biosciences) at RT for 1 h at a dilution of 1/10 000. Membranes were then washed for 10 min in TBST 3 times at RT and imaged with a Li-COR Odyssey. For U2OS and hTERT-RPE1 cells, cells were lysed in Lysis Buffer (150 mM NaCl, 50 mM Tris–HCl, pH7.5, 10% glycerol, 0.5% NP-40, 1 mM MgCl_2_, 1/1000 Benzonase (Sigma), phosphatase (PPI) and protease inhibitors (PI) for 45 min on ice. After pelleting the lysed cells at 14 000 rpm for 15 min at 4°C, total cell extracts (TCE) were collected and the concentration was measured using Bradford Reagent (Sigma). 25 ug of TCE was run on a SDS-PAGE gel at 180 V. Proteins were transferred onto a nitrocellulose membrane for 70 min at 100 V on ice in ice-cold transfer buffer. Membranes were blocked with TBS-T (1× TBS + 0.1% Tween-20) containing 5% milk for 10 min at RT prior to primary antibody incubation overnight. After secondary AB incubation, lane were detected by chemiluminescence on a Vilbur FX6 imager.

### Clonogenic survival assay

For U2OS or hTERT-RPE1 cell lines, cells were trypsinized 48h after siRNA transfection and counted. 500 cells were plated onto a 60 mm dish. For IR sensitivity assays, cells were allowed to adhere for 1 h prior to irradiation at indicated doses (Mainance Millenium Sample Irradiator containing a Cs-137 sealed source), while for Olaparib and ICRF-193 treatments, cells were directly plated in media containing the indicated dose for the duration of the experiment. Cells were grown at 37°C for 10–14 days until colonies were an average of 1–2 mm in diameter. Colonies were stained with DMMB (0.25% dimethylmethylene blue in 50% methanol) and counted.

### Neutral comet assay

Cells were treated with siRNA 48 h prior to IR (3 Gy, Mainance Millenium Sample Irradiator containing a Cs-137 sealed source) and harvested at indicated timepoints. Neutral comet assays were carried out according to manufacturer's guidelines (Trevigen). Briefly, cells were harvested and combined with LMAgarose (Trevigen) at a final concentration of 1 × 10^5^ cells/ml and loaded onto polylysine slides. The agarose plugs were allowed to solidify at 4°C for 1 h before being immersed in lysis buffer (Trevigen) overnight at 4°C. Slides were then equilibrated in cold electrophoresis buffer (100 mM Tris pH9.0, 300 mM NaAz) for 30 min prior to electrophoresis for 1 h at 24 V. The DNA was precipitated for 30 min at RT in DNA precipitation buffer (1 M NH4Ac in EtOH) and washed with 70% EtOH for a further 30 min. Slides were allowed to dry overnight at 37°C prior to staining with SYBR green (Roche, S7563). Images were acquired on a DeltaVision integrated microscope system using the Applied Precision SoftWoRx acquisition software mounted on an IX71 Olympus microscope with a 10× air objective (Imsol). All images were taken as single slices using a CoolSNAP HQ2 ICX-285 CCD camera. Comet analysis was performed using the CometScore software from Tritek Corporation.

### Metaphase spreads

Cells were treated with 2 Gy IR (Xstrahl RS320) and allowed to recover for 48 h. Cells were then captured in metaphase by treating with 200 ng/ml colcemid (Sigma, D7385) for 2 h and were then harvested by trypsinisation as well as their growth medium. Cells were pelleted and resuspended in 10 ml 75 mM potassium chloride and allowed to swell by incubating at 37°C for 30 min. 5 ml of ice-cold fixative (75% methanol, 25% acetic acid) was then slowly added. Cells were then twice pelleted, resuspended in 10 ml fixative and incubated on ice for 2 min. Cells were pelleted and resuspended in 4 ml of fixative, then dropped onto glass slides from a height of 15–20 cm using a p200 pipette. Slides were then steamed over a water bath for 10 s and allowed to dry overnight. The slides were then stained by immersing them in water containing 0.1 μg/ml DAPI and then washed by re-immersing in water and again allowed to dry overnight before coverslips were mounted with Vectashield anti-fade mounting medium (Vector Laboratories, H-1000). Spreads were imaged using a Carl Zeiss LSM 710 confocal microscope and counted manually in ImageJ.

### Cell-cycle assay

For cell cycle analysis, HeLa cells were transfected with siRNA as described above. 48 h post-transfection, cells were trypinised and run through a 40 um cell strainer. Cells were then pelleted at 300 × g for 5min in a swing-out rotor centrifuge, washed once with PBS and resuspended in PBS. Cells were then counted and 5 × 10^5^ cells were used for Flow cytometry analysis. The remaining cells were used to check the extent of siRNA depletion. 5 × 10^5^ cells were then pelleted at 300 × g and resuspended in 300 ul PBS. Under vortex, 700 ul 100% ice-cold ethanol was added to fix the cells dropwise. Fixed cells were then stored at –20°C for 24 h. Cells were then pelleted at 400 × g and the supernatant was decanted. Cells were then briefly vortexed and washed twice with PBS. Cells were then stained with 500 ul PBS containing 250 ug/ml propidium iodide and 40 ug/ml RNaseA) and incubated in the dark for 30 min before analysis on a BD FACS CantoII using BD-FACS DIVA software. Cell populations were gated to exclude doublets and debris and all single cells were then analysed for their PI content.

### Immunofluorescence

Cells were cultured on glass coverslips and either treated with IR, for U2OS, A549 and RPE-1 cells, or with 300nM hydroxytamoxifen (Sigma, H6278) for 4 h, for DIvA cells. Cells were washed once in PBS and then pre-extracted at RT for 3 min in CSK buffer (100 mM NaCl, 300 mM sucrose, 3 mM MgCl_2_, 10 mM PIPES pH 7.0, 50 mM NaF, 5 mM sodium orthovanadate, 10 mM β-glycerol phosphate and 0.7% Triton X-100). Cells were then washed once in CSK buffer, once in PBS and then fixed using 4% paraformaldehyde in PBS for 20 min at RT. Cells were then washed once in PBS, once in TBST and then blocked with TBST containing 10% goat serum (Merck, G9023). Cells were then washed twice in TBST for 5 min at RT and incubated overnight at 4°C with primary antibodies diluted in TBST containing 1% goat serum. All primary antibodies and dilutions are listed in Supplementary Table S1. Cells were then washed 4 times with TBST for 5 min at RT before incubating with Alexa-Fluor conjugated secondary antibodies at RT for 1 h. Cells were then washed 4 times in TBST for 5 min, dipped in water to remove residual buffer and then mounted on glass slides using Vectashield anti-fade hard-set mounting medium containing DAPI (Vector Laboratories, H-1500). Slides were imaged using a Carl Zeiss LSM 710 confocal microscope and images were analysed using the Fiji distribution of ImageJ (35) via the FindFoci plugin (36). FindFoci settings were calibrated on four randomly selected images and these settings were then used to count the foci in all imaged nuclei. For U2OS and hTERT RPE1 WT cells, cells were transfected 48 h prior to treatment. Cells were then fixed with 4% PFA (EMS) for 10 min at RT before permeabilisation with 0.25% Triton-X100 in PBS for 2 min at RT. After blocking with 1% BSA in PBS for 1 h, cells were incubated for 1h with primary antibody at 37°C and subsequent secondary antibody for 1 h at 37°C. Slides were mounted using Vectashield mounting media with DAPI (VectorLaboratories, H1200). Images were acquired on a DeltaVision integrated microscope system using the Applied Precision SoftWoRx acquisition software mounted on an IX71 Olympus microscope with a UPLFLN 40× objective (numerical aperture [NA] 1.3) (Imsol). All images were taken as Z-slices (0.5 μm thickness) using a CoolSNAP HQ2 ICX-285 CCD camera. Parameters for image acquisition were kept constant throughout each experiment. Images were deconvolved using SoftWoRx conservative deconvolution. Quantification was carried out using FIJI software (35). In brief, after deconvolution, the images were projected using ‘sum slices’ (FIJI software) to prevent data loss, and pixel clusters above a defined threshold were counted as individual foci. For representative images, the slices were projected using ‘max projection’.

### DSB reporter assays

For the DR-GFP assays, HR-U2OS cells (Luessing *et al.* 2021, under review) were used, while for the NHEJ (EJ5) U2OS derivative cell line was kindly provided by J. Stark. 2 × 10^6^ cells were transfected with 5 ug pCBA-I-SceI plasmid (Addgene #26477), 40 nmol siRNA and 1 ug Cerulean-c1 plasmid (Addgene #54604) using electroporation (BioRad electroporator). Cells were harvested and resuspended in 500 ul PBS containing 40 nM TOPRO-3 iodide (Life Technologies, #T3605) to identify live cells. The cells were gated for live cells, doublet exclusion and transfected cells (cerulean-positive). A minimum of 20 000 transfected cells were then assessed for the expression of GFP. FACS analysis was carried out using BD FACSCANTOII and BD-FACS DIVA software. The remaining cells were used for checking the knock-down efficiency by western blotting.

### Resection assay

DIvA cells were seeded in six-well plates for 24 h, transfected for 48 h and then treated with 300 nM hydroxytamoxifen for 4 h. Cells were lysed in the wells in cytoplasmic lysis buffer (50 mM HEPES pH7.9, 10 mM KCl_2_, 1.5 mM MgCl_2_, 0.34 M sucrose, 0.5% triton, 10% glycerol, 1 mM DTT) for 10 min on ice, washed once in cytoplasmic lysis buffer and harvested by scraping in genomic extraction buffer (50 mM Tris pH 8.0, 5 mM EDTA pH 8.0, 1% SDS and 0.5 mg/ml proteinase K (Invitrogen, 25530049)). Samples were incubated at 55°C to digest the proteins and the DNA was then ethanol precipitated by adding 0.1 volumes of 3 M sodium acetate pH 5.2 and 2.5 volumes 100% ethanol then incubating on ice for 1 h. The DNA was pelleted at 15 000 × g for 10 min, washed once in 75% ethanol and re-pelleted before air-drying and the resuspending in water. 2 μg of DNA per sample was either digested by BanI or HindIII restriction enzymes or left undigested in separate reactions overnight at 37°C. Samples were diluted to 20 ng/μl and input into qPCR reactions at 40ng/reaction with the primers listed in Supplemental Table S2.

### DNA:RNA-immunoprecipitation (DRIP)

DIvA cells were seeded on 15 cm plates for 24 h, transfected for 48 h and then treated with 300 nM hydroxytamoxifen for 4 h before harvesting with trypsin. Cells were lysed in cytoplasmic lysis buffer (50 mM HEPES pH 7.9, 10 mM KCl_2_, 1.5 mM MgCl_2_, 0.34 M sucrose, 0.5% triton, 10% glycerol, 1 mM DTT) for 10 min on ice and then washed once in cytoplasmic lysis buffer. Nuclei were pelleted at 1500 × g for 5 min at 4°C and then resuspended in genomic extraction buffer (50 mM Tris pH 8.0, 5 mM EDTA pH 8.0, 1% SDS and 0.5 mg/ml proteinase K (Invitrogen, 25530049)) and incubated at 55°C for 1 h to digest all proteins. The genomic DNA was then precipitated by adding 0.1 volumes of 3 M sodium acetate pH 5.2 and then adding 2.5 volumes of 100% ethanol and incubating at RT until the DNA visibly precipitated. The DNA was spooled onto a P1000 pipette tip, washed once in 75% ethanol, air dried and resuspended in 300 ul water. 50 μg of DNA was diluted into 300 ul of water and then sonicated for 10 cycles of 5 s on/30 s off on the low setting in a Diagenode Biorupter and run on a 1.2% agarose gel to ensure fragmentation. 25 ug of fragmented DNA was then equilibrated to 1X RNase-H buffer and treated with 50 units of RNase-H (NEB, M0297L) for 3 h at 37°C, then topped up with an additional 50 units of RNase-H and incubated for a further 3 h. All samples, including RNase-H treated controls, were then equilibrated to 1× DRIP buffer (50 mM Tris pH 8.0, 5mM EDTA pH 8.0, 140 mM NaCl, 1% Triton X-100) and 20 μg was immunoprecipitated overnight at 4°C with 10 μg S9.6 antibody (Merck, MABE1095) conjugated to 100 μl Pierce ChIP-grade protein-A/G magnetic beads (Thermo, 26162). Beads were then washed once in 1× DRIP buffer, once in 1× DRIP buffer with 500 mM NaCl, once in LiCl buffer (10 mM Tris pH 8.0, 250 mM LiCl, 1 mM EDTA pH 8.0, 1% NP-40) and then twice in 1× TE buffer. DNA was eluted from the beads by incubation in 100 μl genomic extraction buffer for 30 min at 55°C. The eluted DNA was then purified via phenol:chloroform:isoamylalcohol (pH 8.0, Sigma P2069) and subsequently ethanol precipitated. The DNA was then either analysed by qPCR using Fast SYBR Green (Thermo, 4385618) or used for a DRIP-seq. qPCR primers used are listed in Supplemental Table S3.

### DRIP-Seq

5 ng of DRIP DNA for two biological replicates was library prepped using the Ultra II DNA library prep kit for Illumina (NEB, E7645L) according to the manufacturer protocol using six PCR cycles. Libraries were then subjected to paired-end 75 cycle sequencing on an Illumina NextSeq 500 an the raw data has been submitted to Array Express. Analysis was done using custom Bash and R scripts, all scripts are available at https://github.com/Bushell-lab/drip_seq. Briefly, fastq files were aligned using Bowtie2 (37) and then sorted and indexed using Samtools (38). Paired alignments were converted to bedpe files using Bedtools and then cut to standard bed files and converted back to bam files. Read coverage was calculated using Samtools Depth and then normalised to total library size. These coverage files were then imported into R for further processing, statistical analysis and plotting.

### Proteomic meta-analysis

Published data from proteomic studies was accessed and analysed using the Damage-Net meta-analysis tool (39). For protein interaction networks, interaction strengths were generated via the STRING database (40) and were then modelled in Cytoscape (41) with groups being created via statistical clustering using the ClusterONE plugin (42).

## RESULTS

**A meta-analysis of proteomic datasets identifies DDX17 as a potentially novel DNA repair factor**

**Figure 1.**
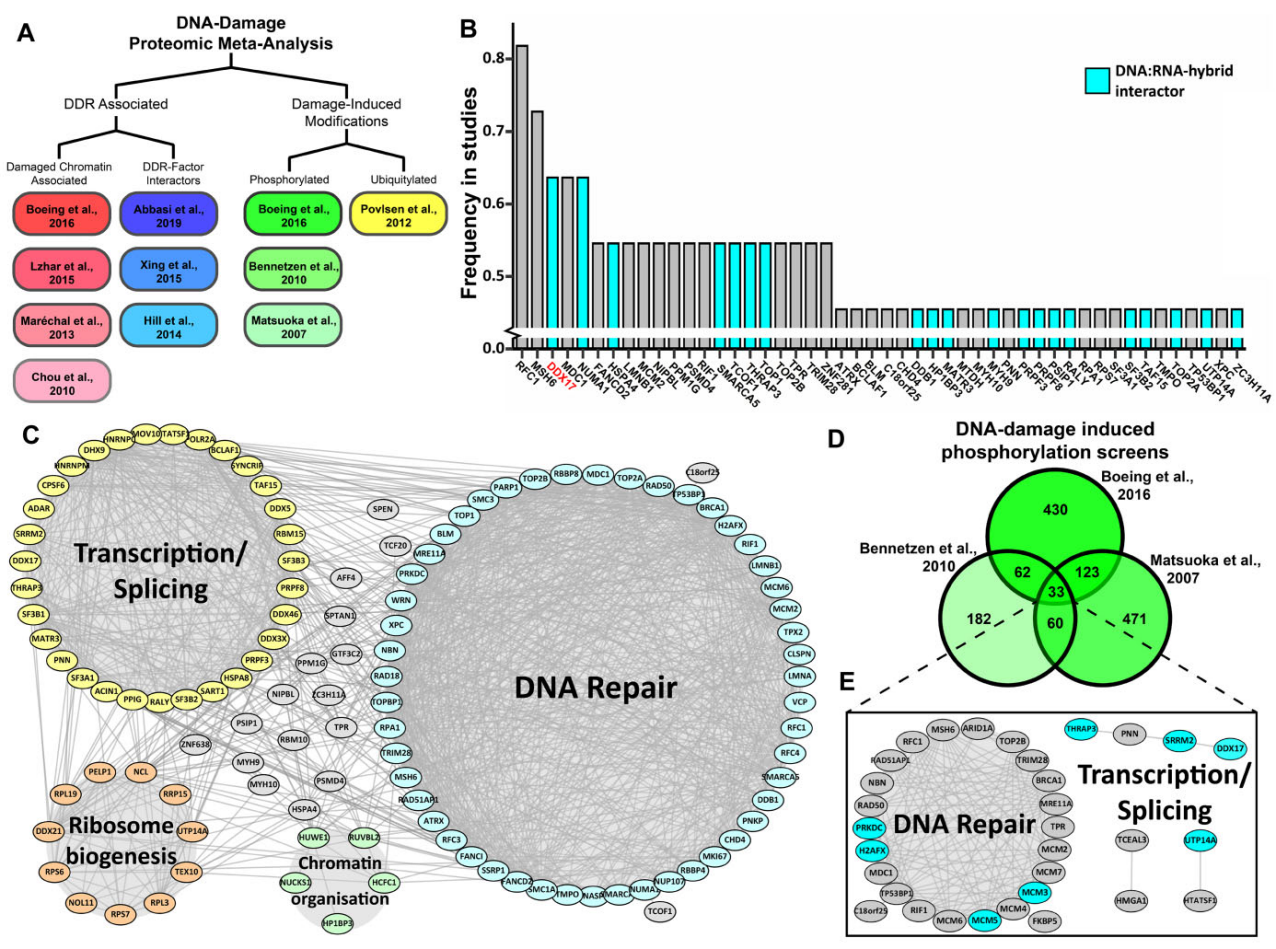
A meta-analysis of proteomic datasets identifies DDX17 as a potentially novel DNA repair factor. (**A**) Design schematic of the proteomic meta-analysis: sub-groups were designed to ensure a broad range of studies were included in the analysis. (**B**) Histogram of the frequency in which proteins were identified by the studies in the meta-analysis with DNA:RNA-hybrid interactors highlighted in cyan, only those identified in five or more studies are shown. (**C**) Protein interaction network of all proteins identified in four or more studies in the meta-analysis. (**D**) Venn diagram of the results of the three phosphoproteomic studies in the meta-analysis. (**E**) Protein interaction network of the 33 central proteins from (D).

Many large-scale proteomic investigations into the DNA-damage response (DDR) have been conducted and a substantial array of such datasets have been published. Therefore, to identify novel DNA repair factors, we conducted a meta-analysis of 11 such proteomic datasets focussed on DNA repair (Supplementary Figure S1A, B) (43–52). Datasets were selected that utilised a variety of experimental approaches, providing a broad set of results that will allow identification of common hits while removing methodology specific effects. This meta-analysis included four major classes of approaches; chromatin association studies (43–46), such as SILAC of chromatin-bound proteins in response to UV damage, DNA-repair factor interactomes (47–49), both AP-MS and Bio-ID of factors such as BRCA1, phosphoproteomics (43,50,51) and ubiquitylomics (52) which were investigating post-translational modifications in response to DNA damage (Figure 1A).

Protein-protein interaction networking of the 119 proteins identified by at least 4 out of the 11 studies in our meta-analysis revealed two major protein clusters; DNA repair and transcription/splicing as well as several other proteins that are partly associated with ribosomal RNA biogenesis and chromatin organisation (Figure 1C). Gene ontology analysis of the 119 enriched genes confirmed this result, as DNA repair and splicing GO terms were among the most significant along with terms associated with DNA replication and chromosome organisation (Supplementary Figure S1C). Ranking all genes by the frequency in which they were identified in these studies again highlighted commonly identified DNA repair factors, such as MDC1 and MSH6. However, some genes which have not been reported to be DNA repair factors were also significantly enriched, such as DDX17 (Figure 1B). Interestingly, several of these enriched genes have also been shown to interact with DNA:RNA-hybrids (53), and DDX17 was found to be the most significantly enriched hybrid interactor without a previously described role in DNA repair (Figure 1B) (54,55).

A further analysis of the phosphoproteomic studies in the meta-analysis found that only 33 genes are shared between all three studies (Figure 1D). The low overlap between these studies is likely due to methodological differences, although those identified in all three studies might be expected to be those most likely to be core DNA repair factors and, as expected, a majority of these 33 proteins are indeed canonical DNA repair factors. However, eight were associated with transcription and splicing processes, DDX17 among them (Figure 1E). Interestingly, all three studies identified the same DDX17 phospho-peptide which harboured 2 ‘SQ’ motifs that are the target of the DDR kinases ATM and ATR (Supplementary Figure S1D). Collectively, this meta-analysis highlighted not only a strong enrichment of transcription and splicing factors in DNA repair processes but, specifically, highlighted DDX17 as a potentially novel DNA repair factor.

### DDX17 is required for cell survival in response to DNA damage

**Figure 2.**
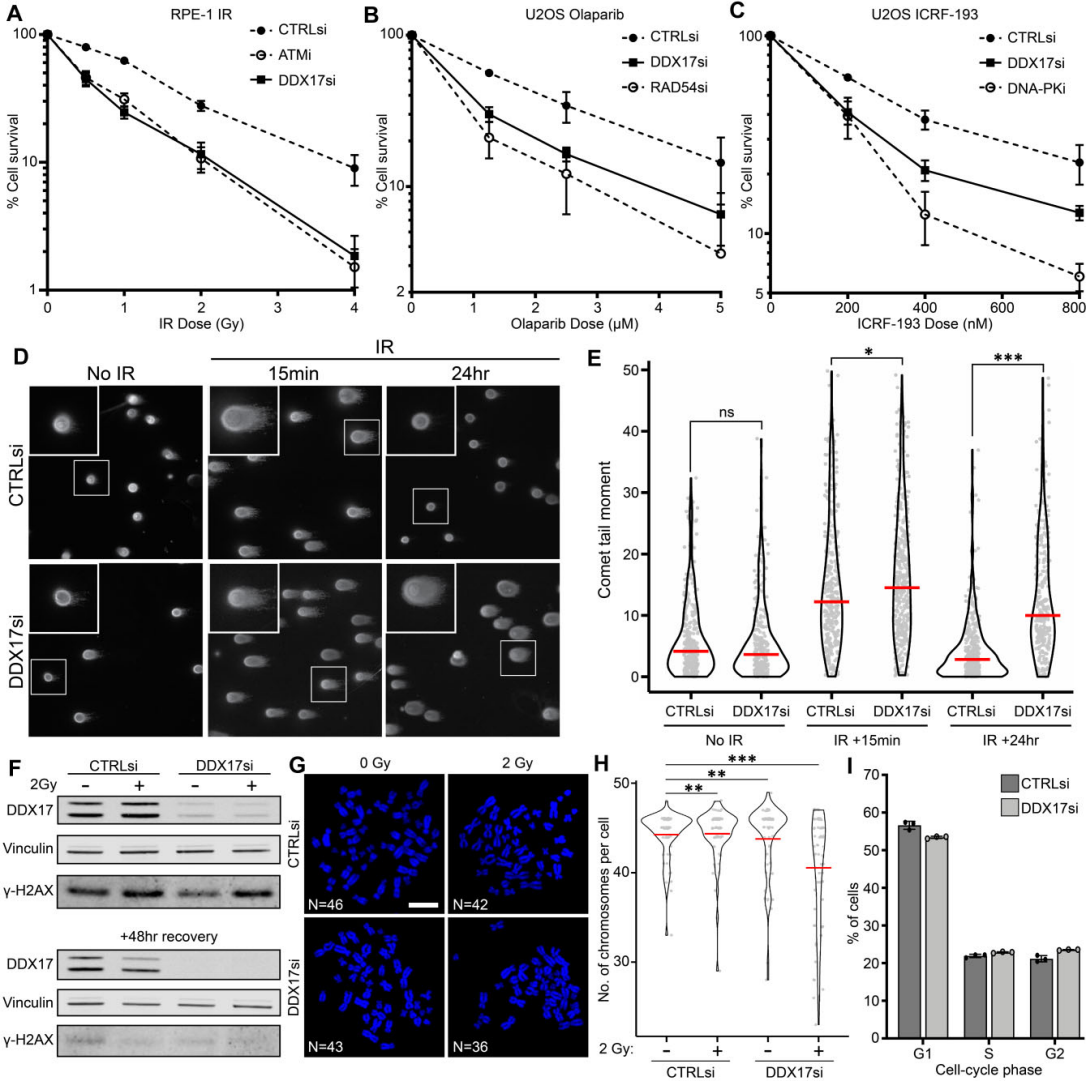
DDX17 is required for cell survival in response to DNA damage. (**A**) Clonogenic assay comparing RPE-1 cell survival in response to increasing doses of IR between the indicated knockdowns and treatments, error bar is SEM of 3 biological replicates. (**B**) Same as (A) but with increasing Olaparib treatment in U2OS cells. (**C**) Same as (A) but with increasing ICRF-193 treatment in U2OS cells. (**D**) Representative images of comet assays with either control and or siRNA with no treatment, 15 min post 3 Gy IR or 24 h post 3 Gy IR. (**E**) Comet tail length quantification plot for (D) of a minimum of 268 cells per condition across two biological replicates, red line is the median. (**F**) Western blot corresponding to metaphase spread experiments in (G). (**G**) Representative images of metaphase spreads treated with either control of DDX17 siRNA and either 0 Gy IR or 2Gy IR. (**H**) Number of chromosomes per cell quantification plot for (G) of a minimum of 60 cells per condition across two biological replicates, scalebar is 5 μM, red line is the mean. (**I**) Cell-cycle analysis results of DDX17 depletion via PI-staining and FACS, bar represents the proportion of the cell population in the indicated cell-cycle phase, dots represent individual biological replicates and bars represent standard deviation.

To investigate this potential role of DDX17 in the DDR, we initially conducted clonogenic survival assays with RPE-1 cells in response to an increasing dose of irradiation (IR) upon siRNA mediated knockdown of DDX17. In RPE-1 cells, DDX17 knockdown resulted in a significant reduction in cell survival in response to as little as 0.5 Gy IR relative to control cells (Figure 2A). This reduction in cell survival was comparable to that observed for treatment with the ATM inhibitor KU55933, suggesting an important role for DDX17 in the DDR. IR sensitivity upon DDX17 depletion was also observed in an independent cell line (Supplementary Figure S2A). These U20S cells were also treated with Olaparib and the topoisomerase II inhibitor ICRF-193, agents specific for HR or NHEJ, respectively (Figure 2B, C). In all of these experiments, DDX17 knockdown was found to significantly reduce cell survival in response to all the DSB-inducing agents used demonstrating a broad role for DDX17 in the DDR. To directly assess the persistence and resolution of DSBs, we next used neutral comet assays. Comet tail moments, the measure of DNA breaks, significantly increased with both control and DDX17 knockdown cells 15 min post 3Gy IR. However, 24 h after irradiation cells in which DDX17 had been depleted had significantly higher comet tail moments than in control cells (Figure 2D, E, Supplementary Figure S2B). This is indicative of a compromised DSB repair and demonstrates that DDX17 depletion significantly reduces DSB repair capacity. To further investigate the direct effect of DDX17 depletion upon maintenance of genome stability, we employed metaphase spreads. DDX17 depleted cells treated with 2 Gy IR and allowed to recover for 2 days (Figure 2F) displayed substantial loss of chromosomes (Figure 2G, H), consistent with sustained, unresolved DNA damage (56,57). Combined with the comet assay results, this provides evidence for the direct involvement of DDX17 in DSB repair and the maintenance of genome stability. To further quantify the role of DDX17 in DSB repair, we conducted repair assays, using the well-established GFP reporter cell lines DR-GFP and EJ5, which quantify the efficiency of HR and NHEJ, respectively. DDX17 depletion significantly reduced the efficiency of both HR and NHEJ repair efficiency, with the NHEJ reporter efficiency being reduced to a level comparable to 53BP1 knockdown (Supplementary Figure S2C, D). Shifts in cell-cycle distribution are known to influence the DNA repair landscape of cells, therefore to assess a potential role of DDX17 in cell-cycle regulation, we conducted a PI-stain cell-cycle assay which found no significant changes in cell-cycle distribution upon DDX17 depletion (Figure 2I).

### DDX17 promotes double-strand break repair factor recruitment

**Figure 3.**
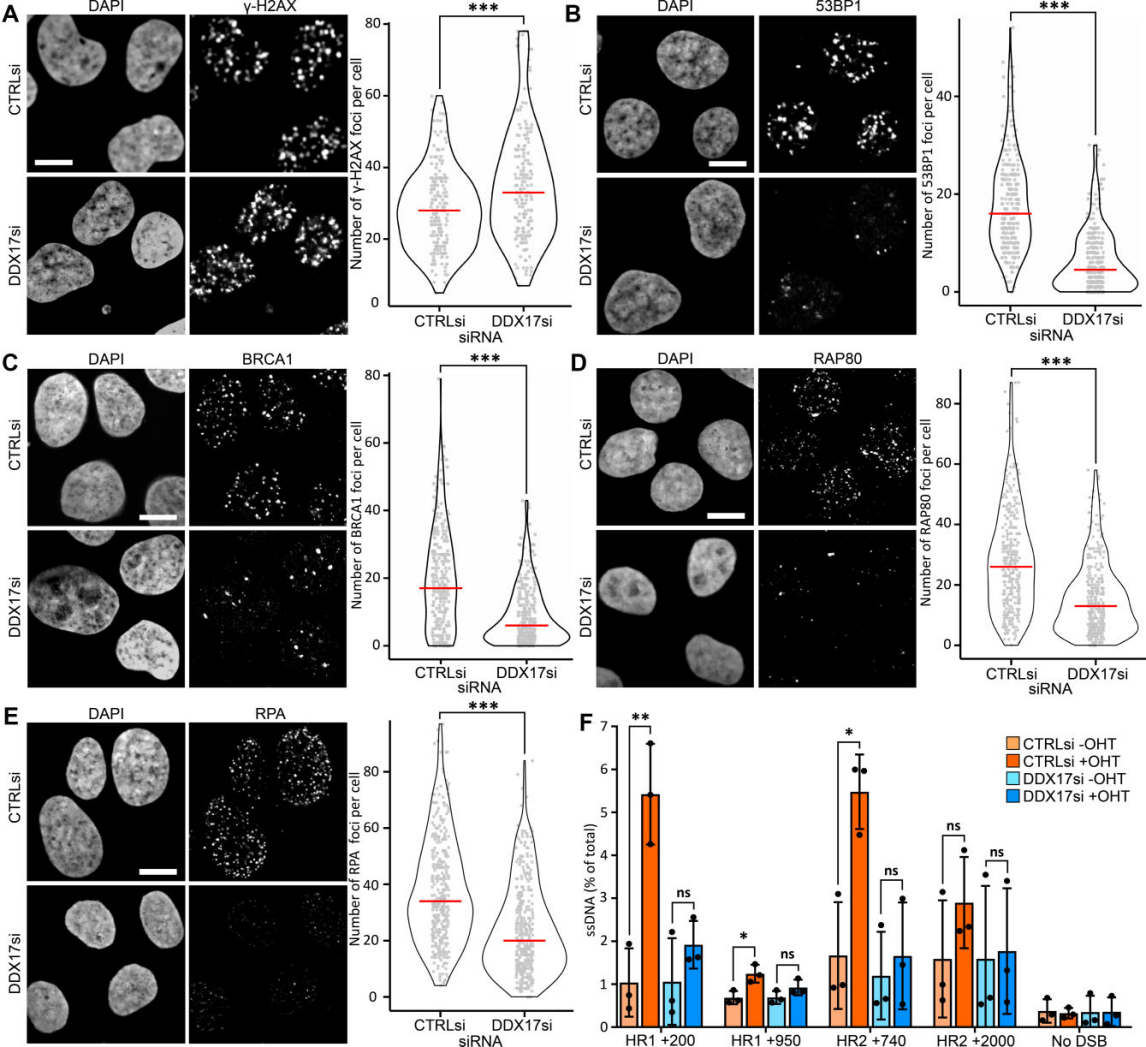
DDX17 promotes double-strand break repair factor recruitment. (**A**) Left: representative images of immunofluorescence of γ-H2AX in DIvA cells treated with 4-hydroxytamoxifen for 4 h with either control or DDX17 siRNA, scalebar is 10μM, right: γ-H2AX foci per cell quantification of a minimum of 250 cells per condition across 3 biological replicates, red line is the median. (**B**) Same as (A) but for 53BP1 immunofluorescence. (**C**) Same as (A) but for BRCA1 immunofluorescence. (**D**) Same as (A) but for RAP80 immunofluorescence. (**E**) Same as (A) but for RPA32 immunofluorescence. All statistics were done using unpaired, directional Wilcoxon tests, *P < 0.05, **P < 0.01, ***P < 0.001. (**F**) Site specific qPCR resection assay at defined distances from two loci, ssDNA quantified relative to input DNA, statistics done using paired *t*-tests *P < 0.05, **P < 0.01.

Given these pronounced DNA repair phenotypes upon DDX17 knockdown, we quantified the focal recruitment of phosphorylated H2AX (γ-H2AX), 53BP1 and BRCA1 using immunofluorescence. For these experiments, we used the ‘Damage-Induced via AsiSI (DIvA)’ cell system pioneered by the Legube lab that uses an AsiSI restriction enzyme fused to an oestrogen receptor to induce double-strand breaks at known genomic loci in response to 4-hydroxytamoxifen (OHT) treatment. DDX17 depletion resulted in a small but significant increase in y-H2AX foci per cell (Figure 3A), again suggesting that DDX17 knockdown results in an accumulation of unresolved DNA damage. However, immunofluorescence of both 53BP1 and BRCA1 showed substantially reduced focal recruitment of both after DDX17 knockdown (Figure 3B, C), which cannot be explained by reduced levels of either 53BP1 or BRCA1 upon DDX17 depletion (Supplementary Figure S3A). We further confirmed these results with IR-induced foci (IRIF) quantifying both γ-H2AX and 53BP1 in U2OS cells. DDX17 depletion once more results in a subtle increase in γ-H2AX IRIF but a significant decrease in 53BP1 foci (Supplementary Figure S3B–D). We next studied the recruitment of RAP80, a member of the BRCA1-A complex that is known to regulate BRCA1 recruitment and resection (58). This similarly found a significant reduction in RAP80 recruitment upon DDX17 depletion (Figure 3D). Examining γ-H2AX, 53BP1 and BRCA1 foci prior to damage induction did not reveal significant changes upon DDX17 depletion (Supplementary Figure S3E–G), indicating that DDX17 does not effect basal DSB levels or repair factor recruitment. In addition, a qPCR assay for DIvA damage induction found no significant change in damage induction with DDX17 depletion (Supplementary Figure S3H). To investigate any potential genetic relationships between DDX17 and 53BP1 or BRCA1, we conducted further clonogenics using olaparib treatment, as 53BP1 is known to complement BRCA1-knockout induced olaparib toxicity (59). Depletion of DDX17 caused olaparib sensitivity in both WT and 53BP1 knockout backgrounds and failed to recover the BRCA1 knockout sensitivity (Supplementary Figure S3I), suggesting DDX17 functions independently of the 53BP1/BRCA1 axis. Given the importance of resection at these early stages of repair, we next studied the effect of DDX17 depletion on resection via the immunofluorescence of RPA which found that DDX17 depletion significantly reduced RPA foci formation (Figure 3E). Furthermore, a qPCR based assay to study resection in the DIvA cell system (60) revealed that even shorter resection of 200bp was significantly reduced by DDX17 depletion (Figure 3F). These results indicate that DDX17 contributes to DDR signalling at a very early stage in the cascade. Thus, our data have identified a crucial role for DDX17 in promoting the recruitment of downstream DSB repair factors to facilitate the repair process and ultimately maintain genome stability.

### Immunofluorescence identifies the ubiquitin cascade as the point of DDX17 action in DSB repair

The recruitment of 53BP1 or BRCA1 to DSBs has been defined as an early event in the response to DSBs that follows a cascade of histone modifications and protein recruitment (6). Therefore, we analysed the recruitment of several components of the cascade to determine DDX17’s point of action.

**Figure 4.**
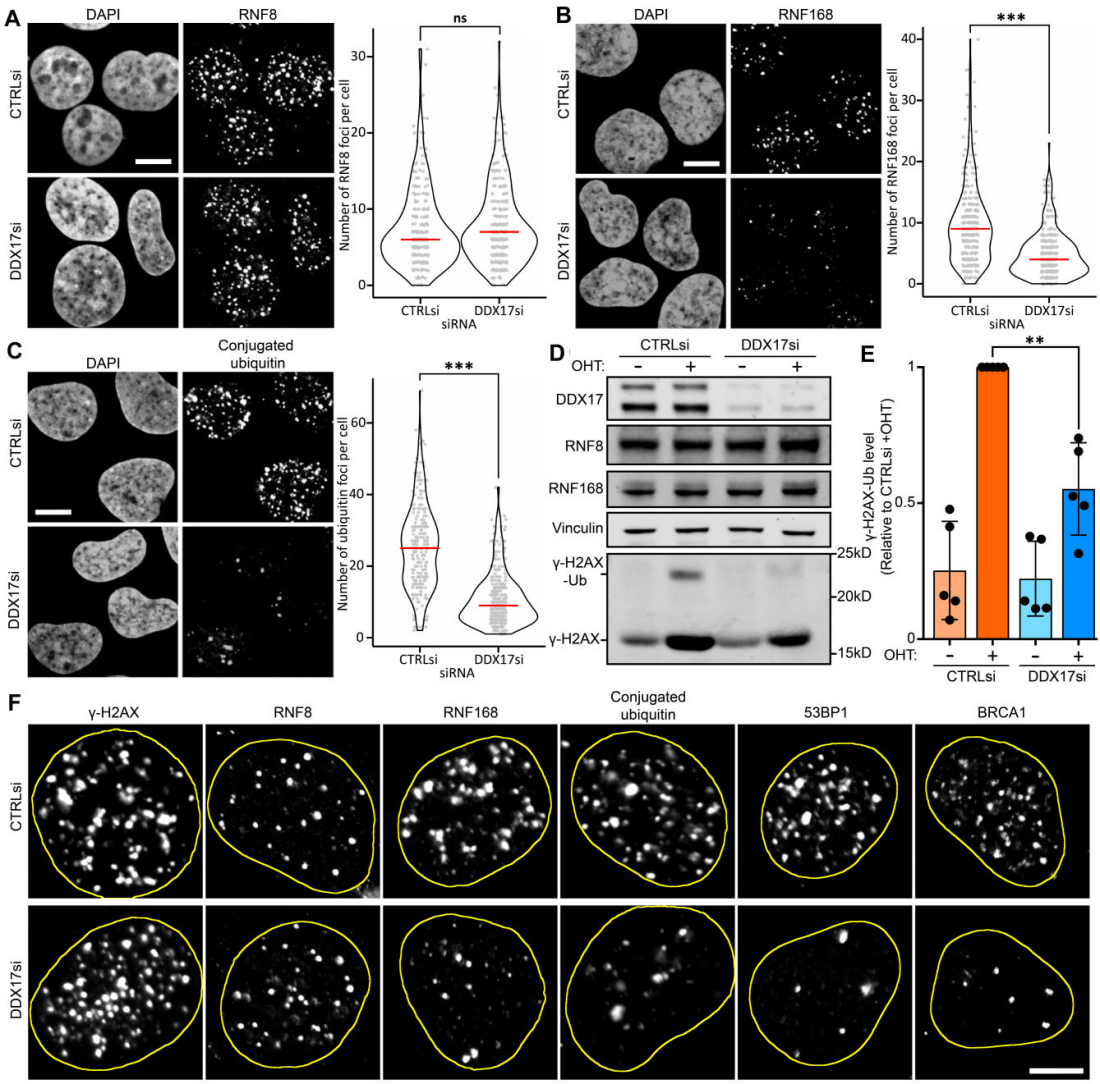
Immunofluorescence identifies the ubiquitin cascade as the point of DDX17 action in DSB repair. (**A**) Left: representative images of immunofluorescence of RNF8 in DIvA cells treated with 4-hydroxytamoxifen for 4 h with either control or DDX17 siRNA, scalebar is 10μM, right: RNF8 foci per cell quantification of a minimum of 265 cells per condition across 3 biological replicates, red line is the median. Statistics done using an unpaired directional Wilcoxon test, not significant (ns). (**B**) Same as (A) but for RNF168 immunofluorescence, ***P < 0.001. (**C**) Same as (A) but for conjugated ubiquitin immunofluorescence, ***P < 0.001. (**D**) Western blots for of DIvA cells transfected with either control or DDX17 siRNA and treated with or without 4-hydroxytamoxifen (OHT) for 4 h, mono-ubiquitylated γ-H2AX is determined by the size shift of the γ-H2AX main band from ∼16 kDa to ∼24 kDa. (**E**) Quantification of γ-H2AX-Ub bands from (D) normalised to loading control then relative to total γ-H2AX and to CTRLsi + OHT. Bar is mean of five biological replicates, points are the individual replicate values, error bar is standard deviation. Statistics were done using a paired *t*-test, **P < 0.01. (**F**) Summary of representative images of immunofluorescence for γ-H2AX, RNF8, RNF168, conjugated ubiquitin, 53BP1 and BRCA1 from the data presented in Figures 3 and 4 with either control or DDX17 siRNA treatment, nuclei are outlined in yellow, scalebar is 5μM.

As our previous immunofluorescence experiments found no decrease in γ-H2AX focus formation upon DDX17 depletion in DIvA cells with induced DSBs (Figure 3A), we next probed for recruitment of the ubiquitin ligase RNF8 which occurs through direct interaction with MDC1. DDX17 depletion did not significantly perturb the formation of RNF8 damage-induced foci (Figure 4A), suggesting a function downstream of RNF8. Recruitment of the subsequent ubiquitin ligase, RNF168, is dependent upon prior RNF8 recruitment and, interestingly, we observed a significant decrease in RNF168 damage-induced foci upon DDX17 depletion (Figure 4B). In addition, the abundance of conjugated ubiquitin, detected with a specific antibody, was also similarly reduced upon DDX17 depletion (Figure 4C). Thus, DDX17 functions downstream of RNF8 to facilitate RNF168 focal recruitment and polyubiquitylation at sites of DNA damage. We used quantitative western blotting of mono-ubiquitylated γ-H2AX, which is dependent upon RNF168 and required for subsequent 53BP1 and BRCA1 recruitment, to support these immunofluorescence data and similarly observed reduced damage-dependent monoubiquitylated γ-H2AX upon DDX17 knockdown (Figure 4D, E). As there is no change in either RNF8 or RNF168 protein levels (Figure 4D), the observed defects in recruitment and ubiquitylation result from regulation of protein recruitment to chromatin proximal to DSBs sites, rather than any changes in gene expression. Consistent with RNF168 activity being required for their recruitment, both 53BP1 and BRCA1 display defective focal recruitment to sites of DNA damage (Figure 4F). We independently validated key results using a different DNA damaging agent and siRNA in A549 cells (Supplementary Figure S4A–D). Thus, DDX17 is required for the recruitment and activity of RNF168, but not RNF8, to chromatin in the vicinity of DSBs, identifying DDX17 as a regulator of efficient DSB signalling that functions between two E3 ubiquitin ligases, RNF8 and RNF168.

### DDX17 promotes double-strand break induced DNA:RNA-hybrids at hybrid deficient genomic loci

Loss of RNF168 recruitment and reduced ubiquitylation at sites of DNA damage is consistent with the phenotype previously observed for the microRNA biogenesis factor DROSHA (13), a known interactor of DDX17 (33,61). This study found DROSHA to be required for the formation of DNA:RNA-hybrids at DSBs, and that these hybrids were critical for effective DSB repair. Given that DDX17 was also previously reported to be a DNA:RNA-hybrid interactor (13), we chose to investigate the effect of DDX17 knockdown on DSB-induced DNA:RNA hybrid formation. To do this, we used the well-established technique of DNA:RNA-hybrid immunoprecipitation (DRIP) in the DIvA cell system to quantify DNA:RNA-hybrid levels around AsiSI-induced DSBs. Western blotting confirmed the expected induction of γ-H2AX and DDX17 depletion (Supplementary Figure S5A).

**Figure 5.**
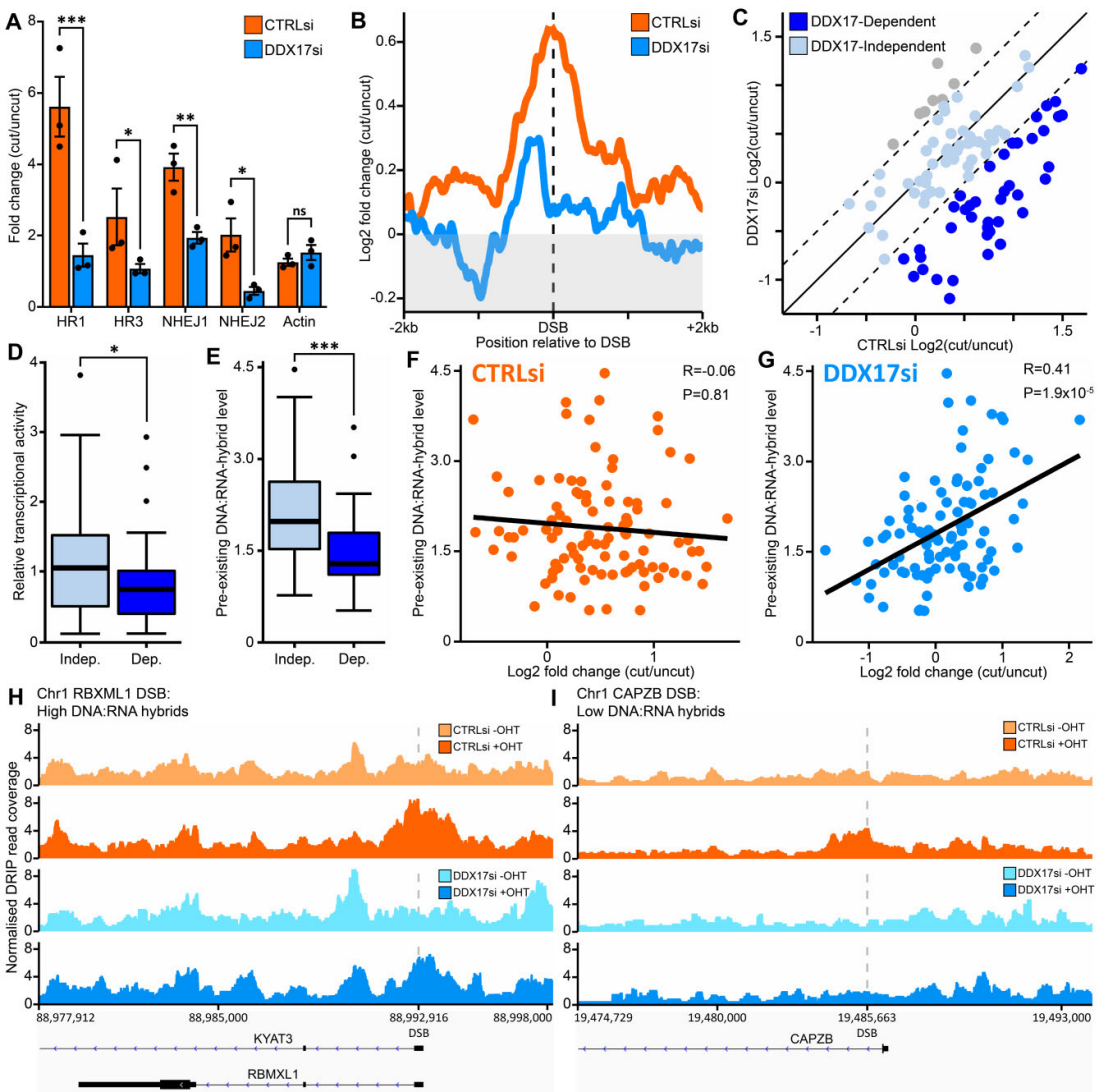
DDX17 promotes double-strand break induced DNA:RNA-hybrids at hybrid deficient genomic loci. (**A**) DNA:RNA IP (DRIP) qPCR around HR and NHEJ repaired DNA break sites in the DIvA cell system and an undamaged site in actin exon 5. Bar represents the mean fold change of damaged (+OHT 4 h) over undamaged (–OHT) for the IP % of input for three biological replicates, error bar is SEM. Statistics were done with a paired *t*-test, *P < 0.05, **P < 0.01, ***P < 0.001. (**B**) DRIP-seq of two of the replicates from (A) DSB centred metagene of 99 loci cut by the AsiSI restriction endonuclease in the DIvA cell system, y-axis is log_2_ fold change of damaged/undamaged normalized readcounts. (**C**) Log_2_ fold change of damaged/undamaged DRIP normalised readcounts at 99 AsiSI cut sites in DDX17 siRNA vs control siRNA treatment. Sites close to the *y* = *x* line (grey blue) are classed as DDX17-independent, the 40 sites deviating from *y* = *x* by less than –0.5 (dark blue) are classed as DDX17-dependent and there are eight sites (grey) that only slightly deviate from *x* = *y* by more than +0.5. (**D**) Boxplot of relative transcriptional activity of the DDX17-independent DSB loci versus the DDX17-dependent DSB loci, statistics are from directional unpaired Wilcoxon test, *P < 0.05. (**E**) Same as (D) but for pre-existing DNA:RNA-hybrid level, ***P < 0.001. (**F**) Correlation plot of pre-existing DNA:RNA-hybrid level at 99 ASISI induced DSB loci against the Log2 fold change of damaged/undamaged DNA:RNA-hybrid level in the control siRNA treated sample, statistics were done with Pearson correlation testing. (**G**) Same as (F) but for the DDX17 siRNA treated sample. (**H**) Genome browser plot of DRIP-seq normalised readcount for ±4 h OHT treated with control siRNA and DDX17 siRNA treated samples at the high pre-existing DNA:RNA-hybrid AsiSI induced DSB locus in the RBMXL1 gene. (**I**) Same as (H) but for the low pre-existing DNA:RNA hybrid level AsiSI induced DSB locus in the CAPZB gene.

As previously reported (13,18,25,62), DRIP-qPCR found that DNA:RNA-hybrid levels increased around the DSB sites in response to induction, but not around an uncut control locus, and that all observed signal was sensitive to RNase-H pre-treatment, indicating that the enrichment is specifically from DNA:RNA-hybrids (Supplementary Figure S5B). Upon DDX17 knockdown, we found that DSB-induced DNA:RNA-hybrids were dramatically reduced at all four loci we quantified by qPCR, suggesting that DDX17 is required for the formation of these structures in response to break formation (Figure 5A, Supplementary Figure S5B). To examine this phenotype at higher resolution, we also conducted high-throughput sequencing of our DRIP samples (i.e. DRIP-Seq), allowing us to analyse the formation of these hybrids across all the sites cut by the AsiSI enzyme in DIvA cells. An overall analysis of the 99 frequently cut AsiSI loci confirmed that DDX17 depletion causes a significant decrease in DSB-induced DNA:RNA-hybrids (Figure 5B, Supplementary Figure S5C). Comparing AsiSI sites previously reported to be prone to either NHEJ or HR repair (63) found a significant decrease in DSB-induced DNA:RNA-hybrids at both groups upon DDX17 knockdown (Supplementary Figure S5D). In addition, comparing DSB sites with high transcriptional activity to those with low transcriptional activity found that DDX17 again significantly reduced DSB-induced DNA:RNA-hybrids in both groups (Supplementary Figure S5E). To delineate the role of DDX17 in RNF168 recruitment and DNA:RNA-hybrid formation, we conducted further DRIP experiments using RNF168 depletion. This found no significant change in DSB-induced DNA:RNA-hybrids with RNF168 loss (Supplementary Figure S5F), indicating that DDX17 driven DNA:RNA-hybrid formation may precede RNF168 recruitment.

A site-by-site analysis showed that DDX17 knockdown only significantly affects a subset of AsiSI DSB sites; approximately half of these sites show little to no change (Figure 5C). This allowed us to categorise the sites into either DDX17-dependent, where DDX17 knockdown reduces DSB-induced hybrid formation, or DDX17-independent, where DDX17 knockdown has no significant effect on DSB-induced hybrid formation (Figure 5C). This classification separates the effects of DDX17 depletion, as the DDX17-dependent sites show no DSB-induced hybrids after DDX17 knockdown whereas the DDX17-independent sites still have a strong induction of DNA:RNA-hybrids levels compared to CTRLsi (Supplementary Figure S5G–I). Classification of these groups allows us to elucidate the factors that contribute to DDX17-dependence of DSB-induced hybrids by comparing the genomic features of these two groups. Additionally, there were eight sites that showed increased DSB-induced hybrids upon DDX17 knockdown (Figure 5C). However, the increase is quite subtle and there are too few of them to accurately investigate the mechanism of break-induced hybrid formation at these sites. We have previously reported that DSB-induced DNA:RNA hybrid formation positively correlates with the pre-existing transcriptional activity of the damaged locus (23). We therefore initially compared the relative transcriptional activity of the DDX17-dependent and independent groups and determined that, although the difference is subtle, DDX17-dependent loci tend to have lower transcriptional activity than the independent sites (Figure 5D). Since there is a subtle relationship between DDX17-dependency and transcriptional activity, we next chose to investigate features related to transcription. We examined the levels of pre-existing DNA:RNA-hybrid between the groups which showed that DDX17-dependent sites have substantially lower levels of pre-existing DNA:RNA-hybrids than DDX17-independent sites (Figure 5E) and this differential was proportionally greater than their decreased relative transcriptional activity.

While it is known that transcriptional activity and DNA:RNA-hybrids are strongly linked, they are not directly proportional due to complex regulatory features governing DNA:RNA-hybrids. Recent studies have shown that DNA and RNA-binding proteins, chromatin modifications, chromatin remodelling and processes such as splicing and replication have significant influence on DNA:RNA-hybrid levels (53,64–69). However, comparing previously published ChIP-seq coverage of 17 different histone modifications between DDX17-dependent and independent loci found only subtle differences in H3K9me2, H3K9me3 and H4S1p between the groups (Supplementary Figure S5J), suggesting that DDX17 functions mostly independent of these modifications. In the presence of DDX17 we found no correlation between pre-existing DNA:RNA-hybrids and those induced upon DNA damage (Figure 5F). However, upon DDX17 depletion we see a dramatic shift resulting in a positive correlation between these features (Figure 5G). Therefore, in the presence of DDX17, DSB-induced hybrids do not rely on pre-existing hybrid levels, but with loss of DDX17 the generation of DSB-induced hybrids becomes dependent on the innate ability of the locus to form these hybrid structures. Interestingly, a re-analysis of our previous DRIP-seq data conducted with *DROSHA* knockdown did not show a significant positive correlation between DSB-induced hybrids and pre-existing hybrids (Supplementary Figure S5K), indicating separate mechanisms for the two proteins. This dependence on DDX17 at sites harbouring low levels of DNA:RNA-hybrids when undamaged can be demonstrated by viewing these features at an individual gene level. For example, when comparing the RBXML1 DSB site which has high pre-existing DNA:RNA hybrids to the CAPZB DSB site which has low pre-existing hybrids (Supplementary Figure S5L). The RBXML1 locus shows DSB-induced DNA:RNA-hybrids in both control and DDX17 knockdowns, whereas the CAPZB locus has little to no induction upon DDX17 knockdown (Figure 5H, I, Supplementary Figure S5M).

We have determined that DDX17 facilitates DSB-induced DNA:RNA-hybrid formation in the vicinity of DSBs and that this effect is most notable at genomic loci with innately low levels of DNA:RNA-hybrids. In fact, DDX17 depletion leads to DSB-induced hybrid levels being directly proportional to the loci's pre-existing hybrid levels, suggesting that without DDX17 this DSB-dependent induction of hybrids relies on the loci's natural ability to form hybrids. As a result, given that these loci are inefficient at forming hybrids normally, we suggest that DDX17-dependent loci require assistance to form DSB-induced DNA:RNA-hybrids, whereas loci of high pre-existing hybrid levels are naturally more efficient at forming DNA:RNA hybrids and are therefore not as dependent upon DDX17.

## DISCUSSION

In this study, we used a meta-analysis of 11 published large-scale proteomic datasets investigating DNA repair mechanisms to identify potentially novel DNA repair factors. A number of these publications noted that RNA related gene groups were significantly enriched in their results (43–45,47,70), and some specifically noted DDX17 as a top hit (47,71). Our analysis yielded a significant number of genes associated with gene expression and splicing, alongside DNA repair genes, as those most commonly identified. In particular, DDX17 was identified with high confidence, as a potential DNA repair gene. Therefore, we chose to investigate the possible role of DDX17 in the DNA damage response.

Clonogenic survival assays revealed that depletion of DDX17 sensitises cells to IR, Olaparib and ICRF-193 indicating a broad role for DDX17 in the response to agents that induce DSBs. Indeed, neutral comet assays found DDX17 depletion to significantly decrease repair of IR-induced DSBs. Consistent with defective DSB repair, metaphase spreads revealed that DDX17 is necessary to prevent chromosomal loss post IR. Thus, DDX17 is important for the maintenance of genome stability.

Localisation of certain DSB repair factors or specific posttranslational modification to focal repair structures demonstrated that while H2AX phosphorylation was not affected by DDX17 depletion, recruitment of both 53BP1, BRCA1 and RAP80 was significantly reduced. Defective recruitment of these regulators of DSB repair pathway choice suggests defective NHEJ and HR pathways of repair, which was confirmed in NHEJ- and HR-specific reporter assays. In addition, end resection was also significantly reduced by the depletion of DDX17. Consistent with a function upstream of 53BP1 and BRCA1, recruitment of RNF168 was significantly DDX17-dependent while RNF8 was DDX17-independent, thus placing the function of DDX17 in signalling DNA damage between these two E3 ubiquitin ligases. As 53BP1 and BRCA1 compete for recruitment to the ubiquitylated H2A and H2AX generated by RNF168, a role for DDX17 in regulating RNF168 can also be inferred from DDX17-dependent formation of conjugated ubiquitin and mono-ubiquitylated γ-H2AX at sites of DNA damage. Competition between 53BP1 and BRCA1 recruitment to RNF168-dependent ubiquitylation of histones is a key regulatory point in the choice between HR and NHEJ and subject to complex regulation (11–13,72–74). It is likely that this stage of the DDR is subject to even more complex regulation than initially believed due to many fine-tuning controls over downstream pathway choice.

DNA:RNA-hybrids are now known to rapidly form at DSBs and are required for efficient repair (13,18,20). Furthermore, several RNA binding proteins having been reported to regulate the formation of DNA:RNA-hybrids around DSBs (13,18,24,25,75). As DDX17 is known to interact with DNA:RNA-hybrids (53), we examined a potential role for DDX17 in regulating DSB-induced DNA:RNA hybrids by quantifying and mapping these hybrids across the genome. We found that DDX17 promotes the formation of DSB-induced DNA:RNA-hybrids and this role is particularly important at loci that normally have low levels of pre-existing DNA:RNA-hybrids. Upon DDX17 depletion, DSB sites in loci with low pre-existing DNA:RNA-hybrids were unable to form the DSB-induced hybrids necessary to propagate an efficient damage signal (13,21,76). However, the role of DDX17 in stimulating these hybrids at loci normally expressing high levels of DNA:RNA hybrids was less important as such loci could form sufficient DSB-induced hybrids even upon depletion of DDX17. We hypothesise that loci with naturally low DNA:RNA-hybrid levels require assistance from DDX17 to efficiently form DSB-induced hybrids as they lack features, such as preferential DNA sequence and chromatin structure (77,78), required for high levels of hybrid formation. As well as RNA helicase activity, DDX17 is known to also possess RNA annealing and branch-migration activity, in which DDX17 was found to be able to exchange strands of a dsRNA molecule with another complementary ssRNA molecule (29). It is possible that DDX17’s role in promoting DSB-induced DNA:RNA-hybrids relates to this function, whereby DDX17 can facilitate the exchange of one dsDNA strand with an ssRNA strand at the DSB to form a hybrid. It is also possible given DDX17’s strong DNA:RNA-hybrid binding capabilities that DDX17 binds to and stabilises these hybrid structures at breaks (53,79). However, further research is needed to fully understand this mechanism, especially given the other DEAD-box helicases Senataxin and DDX5 were found to have the opposite role at DSBs (18,75,80). Since it has been shown previously that these DSB-induced hybrids are necessary for efficient DSB signalling and repair (13,21,76), we propose that DDX17’s role in the DDR is to facilitate the formation of DSB-induced hybrids, a role that is particularly important at loci with normally low levels of DNA:RNA hybrids.

Several canonical DNA repair genes have also been shown to function via RNA and DNA:RNA-hybrid related processes (20,25,81). For example, the HR factor RAD52 was found to facilitate RNA-dependent DNA repair via strand-invasion of RNA into double-stranded DNA to form a DNA:RNA-hybrid (21,81,82). A previous study also reported that the NHEJ complex can associate with DNA:RNA-hybrid at DSBs and that this was necessary for error-free repair (20). Future studies will be required to determine whether DDX17 has functions that interplay with these known DNA repair genes and DNA:RNA-hybrids. However, as DDR ubiquitylation of histones and signalling is DDX17-dependent, it is likely that DDX17-dependent regulation of DNA:RNA hybrids contributes to the remodelling of chromatin flanking DSBs, which is known to be essential for DSB signalling (83–85). This is consistent with the well described ability of DNA:RNA-hybrids to regulate gene expression by recruiting chromatin modifiers to facilitate remodelling and DNA access (86,87). A role for DNA:RNA-hybrids in chromatin remodelling at DSBs to facilitate opening of the chromatin and allowing downstream factor recruitment would explain how the loss of hybrid promoting proteins such as DDX17 and DROSHA impact upon the early DDR, as chromatin decondensation is an early process in the cascade.

We have presented extensive data showing a phenotype of genome instability and inefficient DSB repair in the absence of DDX17. However, further mechanistic studies are needed to uncover DDX17’s specific function in the damage response. Previous reports have found that the loss of factors associated with gene expression can indirectly lead to a genome instability phenotype by altering the expression of DDR genes (88,89). Although we did not find evidence of DDX17 regulating the expression of DDR genes, it is possible that DDX17’s role in the DDR is indirect through the regulation of an additional factor. It should be noted that previous proteomic studies have identified that DDX17 directly interacts with core DSB repair factors such as KU70 (47,90), is recruited to damaged chromatin (46) and is rapidly phosphorylated in response to DNA damage (43,50,51), which suggests a direct role in the DDR.

Elucidating the role of DSB-induced DNA:RNA hybrids in signalling and repairing DNA damage will require identification and full characterisation of all the proteins regulating these structures. The identification of DDX17 as a new contributor to efficient DNA double strand break repair via both major repair pathways, NHEJ and HR, advances our knowledge of the emerging roles for RNA in DNA-repair.

## Supplementary Material

gkac843_Supplemental_File

## Data Availability

DRIP-seq analysis scripts are available at https://github.com/Bushell-lab/drip_seq.

## ACCESSION NUMBERS

DRIP-seq fastq have been deposited with ArrayExpress under the accession E-MTAB-10814.

## References

[1] Ciccia A. , Elledge S.J. The DNA damage response: making it safe to play with knives. Mol. Cell. 2010; 40:179–204. 20965415 10.1016/j.molcel.2010.09.019 PMC2988877

[2] Richardson C. , Jasin M. Frequent chromosomal translocations induced by DNA double-strand breaks. Nature. 2000; 405:697–700. 10864328 10.1038/35015097

[3] Miki Y. , Swensen J., Shattuck-Eidens D., Futreal P.A., Harshman K., Tavtigian S., Liu Q., Cochran C., Bennett L.M., Ding W. A strong candidate for the breast and ovarian cancer susceptibility gene BRCA1. Science. 1994; 266:66–71. 7545954 10.1126/science.7545954

[4] Sandoval N. , Platzer M., Rosenthal A., Dork T., Bendix R., Skawran B., Stuhrmann M., Wegner R.D., Sperling K., Banin S. et al. Characterization of ATM gene mutations in 66 ataxia telangiectasia families. Hum. Mol. Genet. 1999; 8:69–79. 9887333 10.1093/hmg/8.1.69

[5] Blackford A.N. , Jackson S.P ATM, ATR, and DNA-PK: the trinity at the heart of the DNA damage response. Mol. Cell. 2017; 66:801–817. 28622525 10.1016/j.molcel.2017.05.015

[6] Kieffer S.R. , Lowndes N.F. Immediate-early, early, and late responses to DNA double stranded breaks. Front. Genet. 2022; 13:793884. 35173769 10.3389/fgene.2022.793884 PMC8841529

[7] Nowsheen S. , Aziz K., Aziz A., Deng M., Qin B., Luo K., Jeganathan K.B., Zhang H., Liu T., Yu J. et al. L3MBTL2 orchestrates ubiquitin signalling by dictating the sequential recruitment of RNF8 and RNF168 after DNA damage. Nat. Cell Biol. 2018; 20:455–464. 29581593 10.1038/s41556-018-0071-x PMC6083879

[8] Thorslund T. , Ripplinger A., Hoffmann S., Wild T., Uckelmann M., Villumsen B., Narita T., Sixma T.K., Choudhary C., Bekker-Jensen S. et al. Histone H1 couples initiation and amplification of ubiquitin signalling after DNA damage. Nature. 2015; 527:389–393. 26503038 10.1038/nature15401

[9] Schwertman P. , Bekker-Jensen S., Mailand N. Regulation of DNA double-strand break repair by ubiquitin and ubiquitin-like modifiers. Nat. Rev. Mol. Cell Biol. 2016; 17:379–394. 27211488 10.1038/nrm.2016.58

[10] Mattiroli F. , Vissers J.A., van Dijk W., Ikpa P., Citterio E., Vermeulen W., Marteijn J., Sixma T. RNF168 ubiquitinates K13-15 on H2A/H2AX to drive DNA damage signaling. Cell. 2012; 150:1182–1195. 22980979 10.1016/j.cell.2012.08.005

[11] Mandemaker I.K. , van Cuijk L., Janssens R.C., Lans H., Bezstarosti K., Hoeijmakers J.H., Demmers J.A., Vermeulen W., Marteijn J.A. DNA damage-induced histone H1 ubiquitylation is mediated by HUWE1 and stimulates the RNF8-RNF168 pathway. Sci. Rep. 2017; 7:15353. 29127375 10.1038/s41598-017-15194-y PMC5681673

[12] Henriksson S. , Rassoolzadeh H., Hedstrom E., Coucoravas C., Julner A., Goldstein M., Imreh G., Zhivotovsky B., Kastan M.B., Helleday T. et al. The scaffold protein WRAP53beta orchestrates the ubiquitin response critical for DNA double-strand break repair. Genes Dev. 2014; 28:2726–2738. 25512560 10.1101/gad.246546.114 PMC4265676

[13] Lu W.T. , Hawley B.R., Skalka G.L., Baldock R.A., Smith E.M., Bader A.S., Malewicz M., Watts F.Z., Wilczynska A., Bushell M. Drosha drives the formation of DNA:RNA hybrids around DNA break sites to facilitate DNA repair. Nat. Commun. 2018; 9:532. 29416038 10.1038/s41467-018-02893-x PMC5803274

[14] Bader A.S. , Hawley B.R., Wilczynska A., Bushell M. The roles of RNA in DNA double-strand break repair. Br. J. Cancer. 2020; 122:613–623. 31894141 10.1038/s41416-019-0624-1 PMC7054366

[15] Klaric J.A. , Wüst S., Panier S. New faces of old friends: emerging new roles of RNA-binding proteins in the DNA double-strand break response. Front. Mol. Biosci. 2021; 8:385. 10.3389/fmolb.2021.668821 PMC8138124 34026839

[16] Liu S. , Hua Y., Wang J., Li L., Yuan J., Zhang B., Wang Z., Ji J., Kong D. RNA polymerase III is required for the repair of DNA double-strand breaks by homologous recombination. Cell. 2021; 184:1314–1329. 33626331 10.1016/j.cell.2021.01.048

[17] Jungmichel S. , Rosenthal F., Altmeyer M., Lukas J., Hottiger M.O., Nielsen M.L. Proteome-wide identification of poly(ADP-ribosyl)ation targets in different genotoxic stress responses. Mol. Cell. 2013; 52:272–285. 24055347 10.1016/j.molcel.2013.08.026

[18] Cohen S. , Puget N., Lin Y., Clouaire T., Aguirrebengoa M., Rocher V., Pasero P., Canitrot Y., Legube G. Senataxin resolves RNA:DNA hybrids forming at DNA double-strand breaks to prevent translocations. Nat. Commun. 2018; 9:533. 29416069 10.1038/s41467-018-02894-w PMC5803260

[19] Francia S. , Michelini F., Saxena A., Tang D., de Hoon M., Anelli V., Mione M., Carninci P., d’Adda d.F. Site-specific DICER and DROSHA RNA products control the DNA-damage response. Nature. 2012; 488:231. 22722852 10.1038/nature11179 PMC3442236

[20] Chakraborty A. , Tapryal N., Venkova T., Horikoshi N., Pandita R.K., Sarker A.H., Sarkar P.S., Pandita T.K., Hazra T.K. Classical non-homologous end-joining pathway utilizes nascent RNA for error-free double-strand break repair of transcribed genes. Nat. Commun. 2016; 7:13049. 27703167 10.1038/ncomms13049 PMC5059474

[21] Welty S. , Teng Y., Liang Z., Zhao W., Sanders L.H., Greenamyre J.T., Rubio M.E., Thathiah A., Kodali R., Wetzel R. et al. RAD52 is required for RNA-templated recombination repair in post-mitotic neurons. J. Biol. Chem. 2018; 293:1353–1362. 29217771 10.1074/jbc.M117.808402 PMC5787811

[22] Pryde F. , Khalili S., Robertson K., Selfridge J., Ritchie A., Melton D.W., Jullien D., Adachi Y. 53BP1 exchanges slowly at the sites of DNA damage and appears to require RNA for its association with chromatin. J. Cell. Sci. 2005; 118:2043–2055. 15840649 10.1242/jcs.02336

[23] Bader A.S. , Bushell M. DNA:RNA hybrids form at DNA double-strand breaks in transcriptionally active loci. Cell Death. Dis. 2020; 11:280. 32332801 10.1038/s41419-020-2464-6 PMC7181826

[24] Simon N.E. , Yuan M., Kai M. RNA-binding protein RBM14 regulates dissociation and association of non-homologous end joining proteins. Cell Cycle. 2017; 16:1175–1180. 28426349 10.1080/15384101.2017.1317419 PMC5499918

[25] D’Alessandro G. , Whelan D.R., Howard S.M., Vitelli V., Renaudin X., Adamowicz M., Iannelli F., Jones-Weinert C., Lee M., Matti V. et al. BRCA2 controls DNA:RNA hybrid level at DSBs by mediating RNase H2 recruitment. Nat. Commun. 2018; 9:5376. 30560944 10.1038/s41467-018-07799-2 PMC6299093

[26] Moy R.H. , Cole B.S., Yasunaga A., Gold B., Shankarling G., Varble A., Molleston J.M., tenOever B.R., Lynch K.W., Cherry S. Stem-loop recognition by DDX17 facilitates miRNA processing and antiviral defense. Cell. 2014; 158:764–777. 25126784 10.1016/j.cell.2014.06.023 PMC4134512

[27] Lee C.G. RH70, a bidirectional RNA helicase, co-purifies with U1snRNP. J. Biol. Chem. 2002; 277:39679–39683. 12193588 10.1074/jbc.C200337200

[28] Dardenne E. , Polay Espinoza M., Fattet L., Germann S., Lambert M., Neil H., Zonta E., Mortada H., Gratadou L., Deygas M. et al. RNA helicases DDX5 and DDX17 dynamically orchestrate transcription, miRNA, and splicing programs in cell differentiation. Cell Rep. 2014; 7:1900–1913. 24910439 10.1016/j.celrep.2014.05.010

[29] Rössler O.G. , Straka A., Stahl H. Rearrangement of structured RNA via branch migration structures catalysed by the highly related DEAD-box proteins p68 and p72. Nucleic Acids Res. 2001; 29:2088–2096. 11353078 10.1093/nar/29.10.2088 PMC55448

[30] Lamm G.M. , Nicol S.M., Fuller-Pace F., Lamond A.I. p72: a human nuclear DEAD box protein highly related to p68. Nucleic Acids Res. 1996; 24:3739–3747. 8871553 10.1093/nar/24.19.3739 PMC146168

[31] Janknecht R. Multi-talented DEAD-box proteins and potential tumor promoters: p68 RNA helicase (DDX5) and its paralog, p72 RNA helicase (DDX17). Am. J. Transl. Res. 2010; 2:223–234. 20589163 PMC2892403

[32] Lambert M. , Terrone S., Giraud G., Benoit-Pilven C., Cluet D., Combaret V., Mortreux F., Auboeuf D., Bourgeois C.F. The RNA helicase DDX17 controls the transcriptional activity of REST and the expression of proneural microRNAs in neuronal differentiation. Nucleic Acids Res. 2018; 46:7686–7700. 29931089 10.1093/nar/gky545 PMC6125624

[33] Fukuda T. , Yamagata K., Fujiyama S., Matsumoto T., Koshida I., Yoshimura K., Mihara M., Naitou M., Endoh H., Nakamura T. et al. DEAD-box RNA helicase subunits of the drosha complex are required for processing of rRNA and a subset of microRNAs. Nat. Cell Biol. 2007; 9:604–611. 17435748 10.1038/ncb1577

[34] Fortuna T.R. , Kour S., Anderson E.N., Ward C., Rajasundaram D., Donnelly C.J., Hermann A., Wyne H., Shewmaker F., Pandey U.B. DDX17 is involved in DNA damage repair and modifies FUS toxicity in an RGG-domain dependent manner. Acta Neuropathol. 2021; 142:515–536. 34061233 10.1007/s00401-021-02333-z PMC8856901

[35] Schindelin J. , Arganda-Carreras I., Frise E., Kaynig V., Longair M., Pietzsch T., Preibisch S., Rueden C., Saalfeld S., Schmid B. et al. Fiji: an open-source platform for biological-image analysis. Nat. Methods. 2012; 9:676–682. 22743772 10.1038/nmeth.2019 PMC3855844

[36] Herbert A.D. , Carr A.M., Hoffmann E. FindFoci: a focus detection algorithm with automated parameter training that closely matches human assignments, reduces human inconsistencies and increases speed of analysis. PLoS One. 2014; 9:e114749. 25478967 10.1371/journal.pone.0114749 PMC4257716

[37] Langmead B. , Salzberg S.L. Fast gapped-read alignment with bowtie 2. Nat. Methods. 2012; 9:357–359. 22388286 10.1038/nmeth.1923 PMC3322381

[38] Li H. , Handsaker B., Wysoker A., Fennell T., Ruan J., Homer N., Marth G., Abecasis G., Durbin R. 1000 Genome Project Data, Processing Subgroup The sequence alignment/map format and SAMtools. Bioinformatics. 2009; 25:2078–2079. 19505943 10.1093/bioinformatics/btp352 PMC2723002

[39] Bader A.S. , Bushell M. Damage-net: a program for DNA repair meta-analysis identifies a network of novel repair genes that facilitate cancer evolution. DNA Repair (Amst.). 2021; 105:103158. 34147942 10.1016/j.dnarep.2021.103158 PMC8385418

[40] Szklarczyk D. , Gable A.L., Lyon D., Junge A., Wyder S., Huerta-Cepas J., Simonovic M., Doncheva N.T., Morris J.H., Bork P. et al. STRING v11: Protein–protein association networks with increased coverage, supporting functional discovery in genome-wide experimental datasets. Nucleic Acids Res. 2018; 47:D607–D613. 10.1093/nar/gky1131 PMC6323986 30476243

[41] Shannon P. , Markiel A., Ozier O., Baliga N.S., Wang J.T., Ramage D., Amin N., Schwikowski B., Ideker T. Cytoscape: a software environment for integrated models of biomolecular interaction networks. Genome Res. 2003; 13:2498–2504. 14597658 10.1101/gr.1239303 PMC403769

[42] Nepusz T. , Yu H., Paccanaro A. Detecting overlapping protein complexes in protein-protein interaction networks. Nat. Methods. 2012; 9:471–472. 22426491 10.1038/nmeth.1938 PMC3543700

[43] Boeing S. , Williamson L., Encheva V., Gori I., Saunders R.E., Instrell R., Aygün O., Rodriguez-Martinez M., Weems J.C., Kelly G.P. et al. Multiomic analysis of the UV-induced DNA damage response. Cell. Rep. 2016; 15:1597–1610. 27184836 10.1016/j.celrep.2016.04.047 PMC4893159

[44] Izhar L. , Adamson B., Ciccia A., Lewis J., Pontano-Vaites L., Leng Y., Liang A.C., Westbrook T.F., Harper J.W., Elledge S.J. A systematic analysis of factors localized to damaged chromatin reveals PARP-dependent recruitment of transcription factors. Cell. Rep. 2015; 11:1486–1500. 26004182 10.1016/j.celrep.2015.04.053 PMC4464939

[45] Maréchal A. , Li J.M., Ji X.Y., Wu C.S., Yazinski S.A., Nguyen H.D., Liu S., Jiménez A.E., Jin J., Zou L. PRP19 transforms into a sensor of RPA-ssDNA after DNA damage and drives ATR activation via a ubiquitin-mediated circuitry. Mol. Cell. 2014; 53:235–246. 24332808 10.1016/j.molcel.2013.11.002 PMC3946837

[46] Chou D.M. , Adamson B., Dephoure N.E., Tan X., Nottke A.C., Hurov K.E., Gygi S.P., Colaiacovo M.P., Elledge S.J. A chromatin localization screen reveals poly (ADP ribose)-regulated recruitment of the repressive polycomb and NuRD complexes to sites of DNA damage. Proc. Natl. Acad. Sci. U.S.A. 2010; 107:18475–18480. 20937877 10.1073/pnas.1012946107 PMC2972950

[47] Abbasi S. , Schild-Poulter C. Mapping the ku interactome using proximity-dependent biotin identification in human cells. J. Proteome Res. 2019; 18:1064–1077. 30585729 10.1021/acs.jproteome.8b00771

[48] Xing M. , Yang M., Huo W., Feng F., Wei L., Jiang W., Ning S., Yan Z., Li W., Wang Q. et al. Interactome analysis identifies a new paralogue of XRCC4 in non-homologous end joining DNA repair pathway. Nat. Commun. 2015; 6:6233. 25670504 10.1038/ncomms7233 PMC4339890

[49] Hill S.J. , Rolland T., Adelmant G., Xia X., Owen M.S., Dricot A., Zack T.I., Sahni N., Jacob Y., Hao T. et al. Systematic screening reveals a role for BRCA1 in the response to transcription-associated DNA damage. Genes Dev. 2014; 28:1957–1975. 25184681 10.1101/gad.241620.114 PMC4197947

[50] Bennetzen M.V. , Larsen D.H., Bunkenborg J., Bartek J., Lukas J., Andersen J.S. Site-specific phosphorylation dynamics of the nuclear proteome during the DNA damage response. Mol. Cell. Proteomics. 2010; 9:1314–1323. 20164059 10.1074/mcp.M900616-MCP200 PMC2877989

[51] Matsuoka S. , Ballif B.A., Smogorzewska A., McDonald E.R., Hurov K.E., Luo J., Bakalarski C.E., Zhao Z., Solimini N., Lerenthal Y. et al. ATM and ATR substrate analysis reveals extensive protein networks responsive to DNA damage. Science. 2007; 316:1160–1166. 17525332 10.1126/science.1140321

[52] Povlsen L.K. , Beli P., Wagner S.A., Poulsen S.L., Sylvestersen K.B., Poulsen J.W., Nielsen M.L., Bekker-Jensen S., Mailand N., Choudhary C. Systems-wide analysis of ubiquitylation dynamics reveals a key role for PAF15 ubiquitylation in DNA-damage bypass. Nat. Cell Biol. 2012; 14:1089–1098. 23000965 10.1038/ncb2579

[53] Cristini A. , Groh M., Kristiansen M.S., Gromak N. RNA/DNA hybrid interactome identifies DXH9 as a molecular player in transcriptional termination and R-loop-associated DNA damage. Cell. Rep. 2018; 23:1891–1905. 29742442 10.1016/j.celrep.2018.04.025 PMC5976580

[54] Vidi P. , Liu J., Salles D., Jayaraman S., Dorfman G., Gray M., Abad P., Moghe P.V., Irudayaraj J.M., Wiesmüller L. et al. NuMA promotes homologous recombination repair by regulating the accumulation of the ISWI ATPase SNF2h at DNA breaks. Nucleic Acids Res. 2014; 42:6365–6379. 24753406 10.1093/nar/gku296 PMC4041463

[55] Salvador Moreno N. , Liu J., Haas K.M., Parker L.L., Chakraborty C., Kron S.J., Hodges K., Miller L.D., Langefeld C., Robinson P.J. et al. The nuclear structural protein NuMA is a negative regulator of 53BP1 in DNA double-strand break repair. Nucleic Acids Res. 2019; 47:2703–2715. 30812030 10.1093/nar/gkz138 PMC6451129

[56] Dawar S. , Shahrin N.H., Sladojevic N., D’Andrea R., Dorstyn L., Hiwase D.K., Kumar S. Impaired haematopoietic stem cell differentiation and enhanced skewing towards myeloid progenitors in aged caspase-2-deficient mice. Cell Death. Dis. 2016; 7:e2509. 27906175 10.1038/cddis.2016.406 PMC5260989

[57] Karaayvaz-Yildirim M. , Silberman R.E., Langenbucher A., Saladi S.V., Ross K.N., Zarcaro E., Desmond A., Yildirim M., Vivekanandan V., Ravichandran H. et al. Aneuploidy and a deregulated DNA damage response suggest haploinsufficiency in breast tissues of *BRCA2* mutation carriers. Sci. Adv. 2020; 6:eaay2611. 32064343 10.1126/sciadv.aay2611 PMC6989139

[58] Harris J.L. , Khanna K.K. BRCA1 a-complex fine tunes repair functions of BRCA1. Aging. 2011; 3:461–463. 21805697 10.18632/aging.100334 PMC3156597

[59] Bunting S.F. , Callén E., Wong N., Chen H., Polato F., Gunn A., Bothmer A., Feldhahn N., Fernandez-Capetillo O., Cao L. et al. 53BP1 inhibits homologous recombination in Brca1-deficient cells by blocking resection of DNA breaks. Cell. 2010; 141:243–254. 20362325 10.1016/j.cell.2010.03.012 PMC2857570

[60] Zhou Y. , Caron P., Legube G., Paull T.T. Quantitation of DNA double-strand break resection intermediates in human cells. Nucleic Acids Res. 2014; 42:e19. 24362840 10.1093/nar/gkt1309 PMC3919611

[61] Gregory R.I. , Yan K., Amuthan G., Chendrimada T., Doratotaj B., Cooch N., Shiekhattar R. The microprocessor complex mediates the genesis of microRNAs. Nature. 2004; 432:235–240. 15531877 10.1038/nature03120

[62] Burger K. , Schlackow M., Gullerova M. Tyrosine kinase c-abl couples RNA polymerase II transcription to DNA double-strand breaks. Nucleic Acids Res. 2019; 47:3467–3484. 30668775 10.1093/nar/gkz024 PMC6468493

[63] Aymard F. , Bugler B., Schmidt C.K., Guillou E., Caron P., Briois S., Iacovoni J.S., Daburon V., Miller K.M., Jackson S.P. et al. Transcriptionally active chromatin recruits homologous recombination at DNA double-strand breaks. Nat. Struct. Mol. Biol. 2014; 21:366–374. 24658350 10.1038/nsmb.2796 PMC4300393

[64] Bayona-Feliu A. , Casas-Lamesa A., Reina O., Bernués J., Azorín F. Linker histone H1 prevents R-loop accumulation and genome instability in heterochromatin. Nat. Commun. 2017; 8:283. 28819201 10.1038/s41467-017-00338-5 PMC5561251

[65] Castellano-Pozo M. , Santos-Pereira J.M., Rondón A.G., Barroso S., Andújar E., Pérez-Alegre M., García-Muse T., Aguilera A. R loops are linked to histone H3 S10 phosphorylation and chromatin condensation. Mol. Cell. 2013; 52:583–590. 24211264 10.1016/j.molcel.2013.10.006

[66] Nguyen D.T. , Voon H.P.J., Xella B., Scott C., Clynes D., Babbs C., Ayyub H., Kerry J., Sharpe J.A., Sloane-Stanley J.A. et al. The chromatin remodelling factor ATRX suppresses R-loops in transcribed telomeric repeats. EMBO Rep. 2017; 18:914–928. 28487353 10.15252/embr.201643078 PMC5452009

[67] Chang E.Y. , Novoa C.A., Aristizabal M.J., Coulombe Y., Segovia R., Chaturvedi R., Shen Y., Keong C., Tam A.S., Jones S.J.M. et al. RECQ-like helicases sgs1 and BLM regulate R-loop-associated genome instability. J. Cell Biol. 2017; 216:3991–4005. 29042409 10.1083/jcb.201703168 PMC5716281

[68] Hegazy Y.A. , Fernando C.M., Tran E.J. The balancing act of R-loop biology: the good, the bad, and the ugly. J. Biol. Chem. 2020; 295:905–913. 31843970 10.1074/jbc.REV119.011353 PMC6983857

[69] Kim S. , Kang N., Park S.H., Wells J., Hwang T., Ryu E., Kim B., Hwang S., Kim S., Kang S. et al. ATAD5 restricts R-loop formation through PCNA unloading and RNA helicase maintenance at the replication fork. Nucleic Acids Res. 2020; 48:7218–7238. 32542338 10.1093/nar/gkaa501 PMC7367208

[70] Beli P. , Lukashchuk N., Wagner S.A., Weinert B.T., Olsen J.V., Baskcomb L., Mann M., Jackson S.P., Choudhary C. Proteomic investigations reveal a role for RNA processing factor THRAP3 in the DNA damage response. Mol. Cell. 2012; 46:212–225. 22424773 10.1016/j.molcel.2012.01.026 PMC3565437

[71] Adamson B. , Smogorzewska A., Sigoillot F.D., King R.W., Elledge S.J. A genome-wide homologous recombination screen identifies the RNA-binding protein RBMX as a component of the DNA-damage response. Nat. Cell Biol. 2012; 14:318–328. 22344029 10.1038/ncb2426 PMC3290715

[72] Tang J. , Cho N.W., Cui G., Manion E.M., Shanbhag N.M., Botuyan M.V., Mer G., Greenberg R.A. Acetylation limits 53BP1 association with damaged chromatin to promote homologous recombination. Nat. Struct. Mol. Biol. 2013; 20:317–325. 23377543 10.1038/nsmb.2499 PMC3594358

[73] Jacquet K. , Fradet-Turcotte A., Avvakumov N., Lambert J., Roques C., Pandita R.K., Paquet E., Herst P., Gingras A., Pandita T.K. et al. The TIP60 complex regulates bivalent chromatin recognition by 53BP1 through direct H4K20me binding and H2AK15 acetylation. Mol. Cell. 2016; 62:409–421. 27153538 10.1016/j.molcel.2016.03.031 PMC4887106

[74] Walser F. , Mulder M.P.C., Bragantini B., Burger S., Gubser T., Gatti M., Botuyan M.V., Villa A., Altmeyer M., Neri D. et al. Ubiquitin phosphorylation at thr12 modulates the DNA damage response. Mol. Cell. 2020; 80:423–436. 33022275 10.1016/j.molcel.2020.09.017 PMC7655664

[75] Yu Z. , Mersaoui S.Y., Guitton-Sert L., Coulombe Y., Song J., Masson J., Richard S. DDX5 resolves R-loops at DNA double-strand breaks to promote DNA repair and avoid chromosomal deletions. NAR Cancer. 2020; 2:zcaa028. 33015627 10.1093/narcan/zcaa028 PMC7520851

[76] Garcia-Rubio M.L. , Perez-Calero C., Barroso S.I., Tumini E., Herrera-Moyano E., Rosado I.V., Aguilera A. The fanconi anemia pathway protects genome integrity from R-loops. PLoS Genet. 2015; 11:e1005674. 26584049 10.1371/journal.pgen.1005674 PMC4652862

[77] Chédin F. Nascent connections: R-loops and chromatin patterning. Trends in Genetics : TIG. 2016; 32:828–838. 27793359 10.1016/j.tig.2016.10.002 PMC5123964

[78] Chen L. , Chen J., Zhang X., Gu Y., Xiao R., Shao C., Tang P., Qian H., Luo D., Li H. et al. R-ChIP using inactive RNase h reveals dynamic coupling of R-loops with transcriptional pausing at gene promoters. Mol. Cell. 2017; 68:745–757. 29104020 10.1016/j.molcel.2017.10.008 PMC5957070

[79] Mosler T. , Conte F., Longo G.M.C., Mikicic I., Kreim N., Möckel M.M., Petrosino G., Flach J., Barau J., Luke B. et al. R-loop proximity proteomics identifies a role of DDX41 in transcription-associated genomic instability. Nat. Commun. 2021; 12:7314. 34916496 10.1038/s41467-021-27530-y PMC8677849

[80] Sessa G. , Gómez-González B., Silva S., Pérez-Calero C., Beaurepere R., Barroso S., Martineau S., Martin C., Ehlén Å, Martínez J.S. et al. BRCA2 promotes DNA-RNA hybrid resolution by DDX5 helicase at DNA breaks to facilitate their repair‡. EMBO J. 2021; 40:e106018. 33634895 10.15252/embj.2020106018 PMC8013831

[81] Mazina O.M. , Keskin H., Hanamshet K., Storici F., Mazin A.V. Rad52 inverse strand exchange drives RNA-templated DNA double-strand break repair. Mol. Cell. 2017; 67:19–29. 28602639 10.1016/j.molcel.2017.05.019 PMC5547995

[82] Yasuhara T. , Kato R., Hagiwara Y., Shiotani B., Yamauchi M., Nakada S., Shibata A., Miyagawa K. Human rad52 promotes XPG-mediated R-loop processing to initiate transcription-associated homologous recombination repair. Cell. 2018; 175:558–570. 30245011 10.1016/j.cell.2018.08.056

[83] Ziv Y. , Bielopolski D., Galanty Y., Lukas C., Taya Y., Schultz D.C., Lukas J., Bekker-Jensen S., Bartek J., Shiloh Y. Chromatin relaxation in response to DNA double-strand breaks is modulated by a novel ATM- and KAP-1 dependent pathway. Nat. Cell Biol. 2006; 8:870. 16862143 10.1038/ncb1446

[84] Price B.D. , D’Andrea A.D Chromatin remodeling at DNA double strand breaks. Cell. 2013; 152:1344–1354. 23498941 10.1016/j.cell.2013.02.011 PMC3670600

[85] Clouaire T. , Rocher V., Lashgari A., Arnould C., Aguirrebengoa M., Biernacka A., Skrzypczak M., Aymard F., Fongang B., Dojer N. et al. Comprehensive mapping of histone modifications at DNA double-strand breaks deciphers repair pathway chromatin signatures. Mol. Cell. 2018; 72:250–262. 30270107 10.1016/j.molcel.2018.08.020 PMC6202423

[86] Skourti-Stathaki K. , Proudfoot N.J. A double-edged sword: r loops as threats to genome integrity and powerful regulators of gene expression. Genes Dev. 2014; 28:1384–1396. 24990962 10.1101/gad.242990.114 PMC4083084

[87] Niehrs C. , Luke B. Regulatory R-loops as facilitators of gene expression and genome stability. Nat. Rev. Mol. Cell Biol. 2020; 21:167–178. 32005969 10.1038/s41580-019-0206-3 PMC7116639

[88] Pederiva C. , Böhm S., Julner A., Farnebo M. Splicing controls the ubiquitin response during DNA double-strand break repair. Cell Death Differ. 2016; 23:1648. 27315300 10.1038/cdd.2016.58 PMC5041194

[89] Lee S. , Kopp F., Chang T., Sataluri A., Chen B., Sivakumar S., Yu H., Xie Y., Mendell J.T. Noncoding RNA NORAD regulates genomic stability by sequestering PUMILIO proteins. Cell. 2016; 164:69–80. 26724866 10.1016/j.cell.2015.12.017 PMC4715682

[90] Findlay S. , Heath J., Luo V.M., Malina A., Morin T., Coulombe Y., Djerir B., Li Z., Samiei A., Simo-Cheyou E. et al. SHLD2/FAM35A co-operates with REV7 to coordinate DNA double-strand break repair pathway choice. EMBO J. 2018; 37:e100158. 30154076 10.15252/embj.2018100158 PMC6138439

