# Correction to ‘DDX17 is required for efficient DSB repair at DNA: RNA hybrid deficient loci’

**DOI:** 10.1093/nar/gkad771

**Published:** 2023-09-15

*Nucleic Acids Research*, Volume 50, Issue 18, 14 October 2022, Pages 10487–10502, https://doi.org/10.1093/nar/gkac843

In the originally published version of this manuscript, the representative images for control siRNA and DDX17 siRNA are swapped around in Figure 3A. The corrected version of Figure 3 is included here, and to avoid further confusion the authors have chosen different cells for control siRNA in this panel so that the image no longer appears too be duplicated.

**Figure 3.**
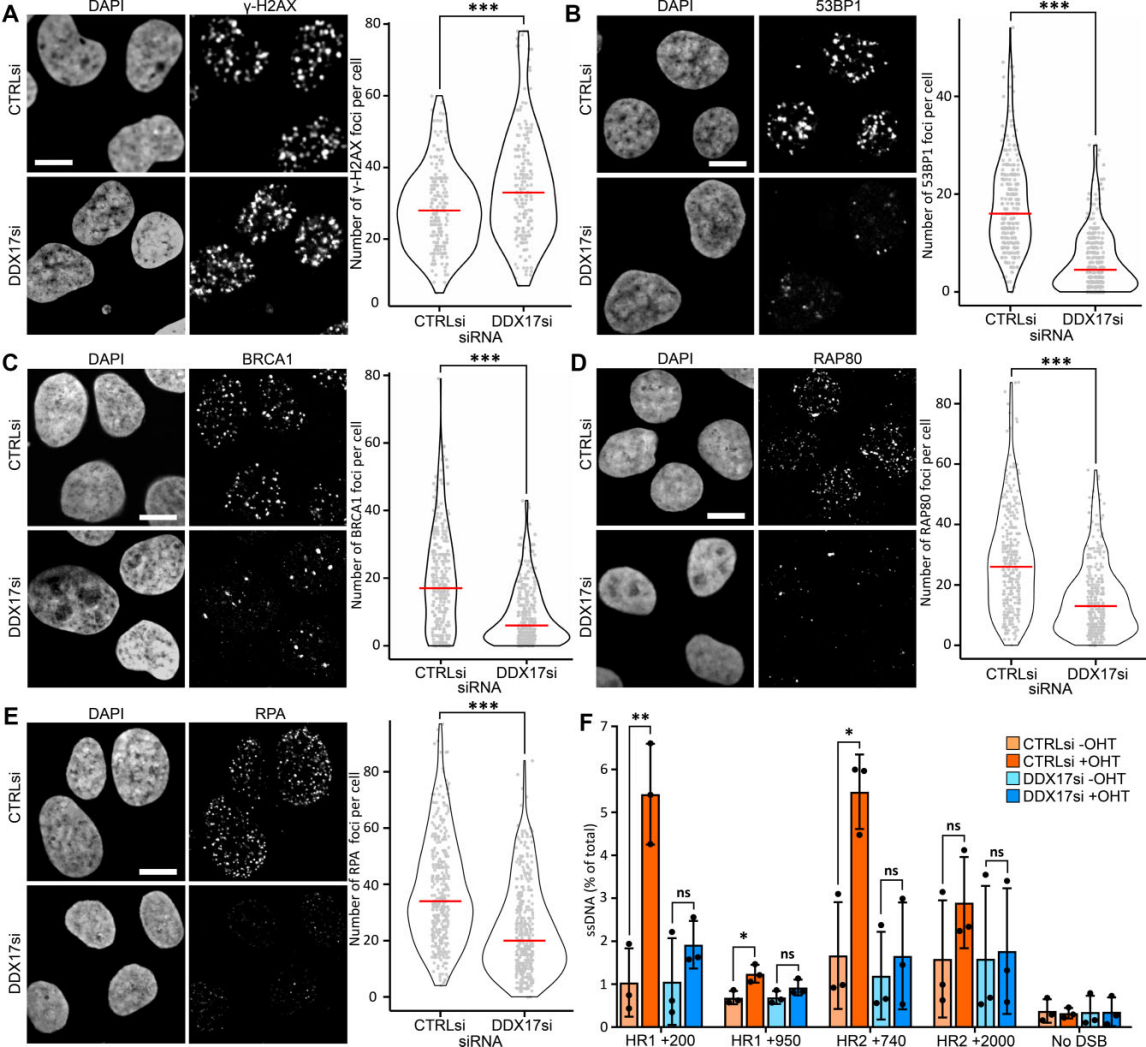
DDX17 promotes double-strand break repair factor recruitment. (**A**) Left: representative images of immunofluorescence of γ-H2AX in DIvA cells treated with 4-hydroxytamoxifen for 4 h with either control or DDX17 siRNA, scalebar is 10μM, right: γ-H2AX foci per cell quantification of a minimum of 250 cells per condition across 3 biological replicates, red line is the median. (**B**) Same as (A) but for 53BP1 immunofluorescence. (**C**) Same as (A) but for BRCA1 immunofluorescence. (**D**) Same as (A) but for RAP80 immunofluorescence. (**E**) Same as (A) but for RPA32 immunofluorescence. All statistics were done using unpaired, directional Wilcoxon tests, *P < 0.05, **P < 0.01, ***P < 0.001. (**F**) Site specific qPCR resection assay at defined distances from two loci, ssDNA quantified relative to input DNA, statistics done using paired *t*-tests *P < 0.05, **P < 0.01.

This error has been corrected online.

