# Supplementary material for: DDX17 is required for efficient DSB repair at DNA:RNA hybrid deficient loci
Source: Nucleic Acids Res. 2022 Oct 6;50(18):10487–502. doi: 10.1093/nar/gkac843 (PMC9561282; doi:10.1093/nar/gkac843)
Supplement: gkac843_Supplemental_File [file gkac843_supplemental_file.pdf]

A

Study	Group	Target	Damage type	Method
Boeing et al., 2016	Damaged Chromatin Associated	Chromatin associated proteins	UV - 30 J/m^2	SILAC
Izhar et al., 2015	Damaged Chromatin Associated	γ-H2AX colocalization	UV micro-irradiation	Immunofluorescence
Maréchal et al., 2013	Damaged Chromatin Associated	RPA-ssDNA associated	None	In-vitro RPA-ssDNA-Biotin
Chou et al., 2010	Damaged Chromatin Associated	Chromatin associated proteins	UV - 50 J/m^2	SILAC
Abbasi et al., 2019	DDR-Factor Interactors	KU70 Interactome	None	BioID
Xing et al., 2015	DDR-Factor Interactors	NHEJ-complex interactome	None	AP-MS
Hill et al., 2014	DDR-Factor Interactors	BRCA1 interactome	None	TAP-MS
Boeing et al., 2016	Phosphorylated	Phosphoproteomics	UV - 30 J/m^2	SILAC
Bennetzen et al., 2010	Phosphorylated	Phosphoproteomics	IR - 6 Gy	SILAC
Matsuoka et al., 2007	Phosphorylated	Phosphoproteomics	IR - 10 Gy	“pS/pT-Q” IP SILAC
Povlsen et al., 2012	Ubiquitylated	Ubiquitylomics	UV - 40 J/m^2	SILAC

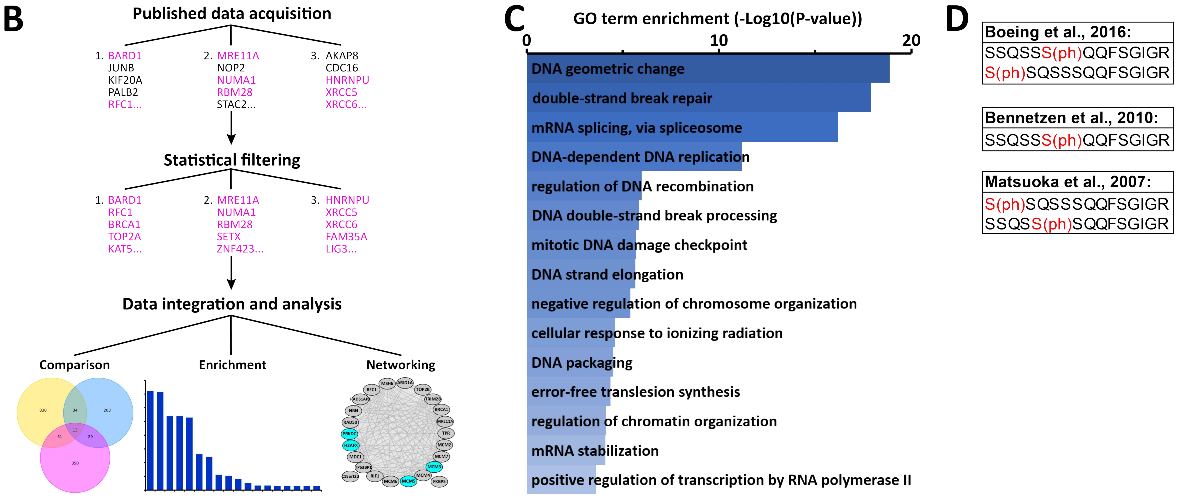

Supplementary Figure 1: Design of a meta-analysis of proteomic datasets to identify novel DNA repair factors. **(A)** Table of the 11 studies included in the meta-analysis and information related to them. **(B)** Workflow for the meta-analysis: Results work accessed from publications, filtered for significant results and then integrated into various analyses. **(C)** Gene ontology enrichment analysis of all genes identified in 4 or of the studies in the meta-analysis. **(D)** DDX17 phosphopeptides identified by the three phosphoproteomic studies in the meta-analysis.

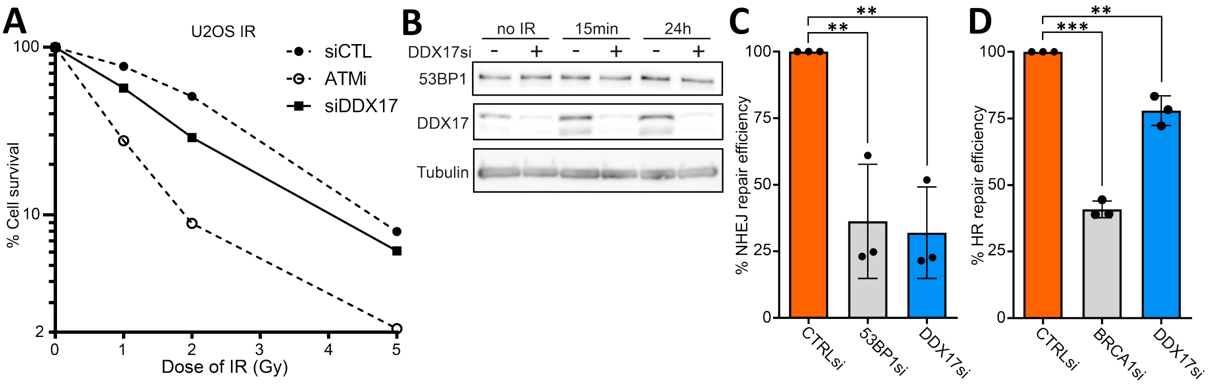

Supplementary Figure 2: DDX17 is required for cell survival in response to DNA damage. **(A)** Clonogenic assay comparing U2OS cell survival in response to increasing doses of IR between the indicated knockdowns and treatments, error bar is SEM across 3 biological replicates. **(B)** Western blot of U2OS cells used in comet assays, treated with either control or DDX17 siRNA and with 0Gy or 3Gy IR followed by 15 minutes or 24 hours. **(C)** EJ5 NHEJ repair reporter assay for 53BP1 and DDX17 knockdowns. Bar height is mean of replicates, points are individual replicate values, error bar is standard deviation, statistics were done using paired t-test, **p<0.01. **(D)** Same as (C) but for the DR-GFP HR repair reporter assay with BRCA1 and DDX17 knockdowns, ***p<0.001.

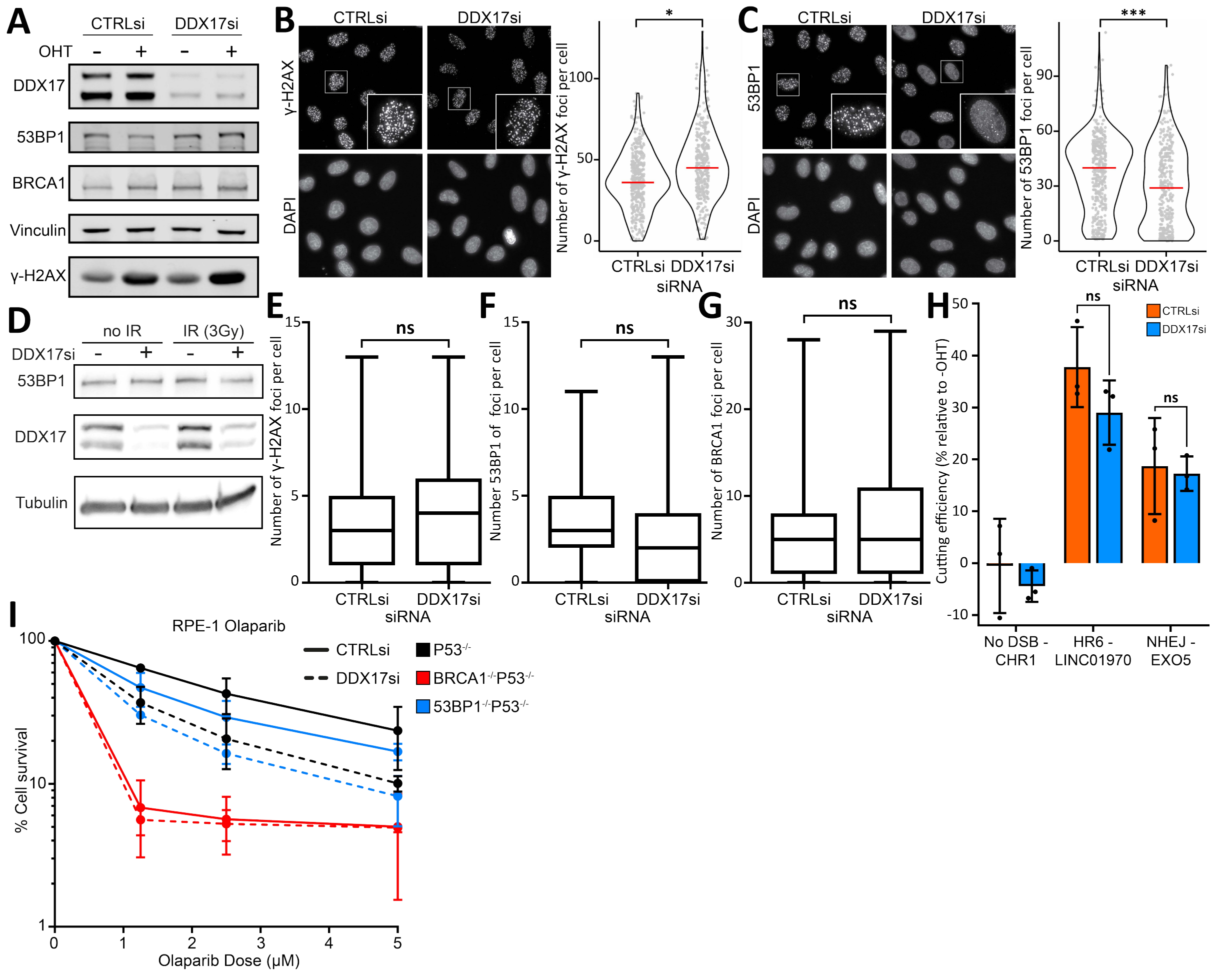

Supplemental Figure 3: DDX17 is required for RNF168 recruitment and histone ubiquitylation at DSBs. **(A)** Western blots of DIvA cells transfected with either control or DDX17 siRNA and treated with or without 4-hydroxytamoxifen (OHT). **(B)** Left: representative images of immunofluorescence of γ-H2AX in U2OS cells treated with 3Gy IR with either control or DDX17 siRNA, right: γ-H2AX foci per cell quantification of a minimum of 340 cells per condition across 3 biological replicates, red line is the median. Statistics done using an unpaired directional Wilcoxon test, *p<0.05. **(C)** same as (B) but for 53BP1 immunofluorescence, ***p<0.001. **(D)** Western blot of conditions from (A-B) treated with either control DDX17 siRNA and 0Gy or 3Gy. **(E)** γ-H2AX foci per cell quantification via immunofluorescence for untreated DIvA cells, minimum of 100 cells quantified across 3 biological replicates for each condition. **(F)** Same as (E) but for 53BP1 immunofluorescence. **(G)** Same as (E) but for BRCA1 immunofluorescence. **(H)** Site specific qPCR cleavage assay at 3 loci; one HR-prone, one NHEJ-prone and one uncut control locus, quantified relative to uncut sample, statistics done using paired t-tests. **(I)** Clonogenic assay comparing survival in response to increasing doses of olaparib between the indicated knockout cell lines and treatments, error bar is SEM across 3 biological replicates.

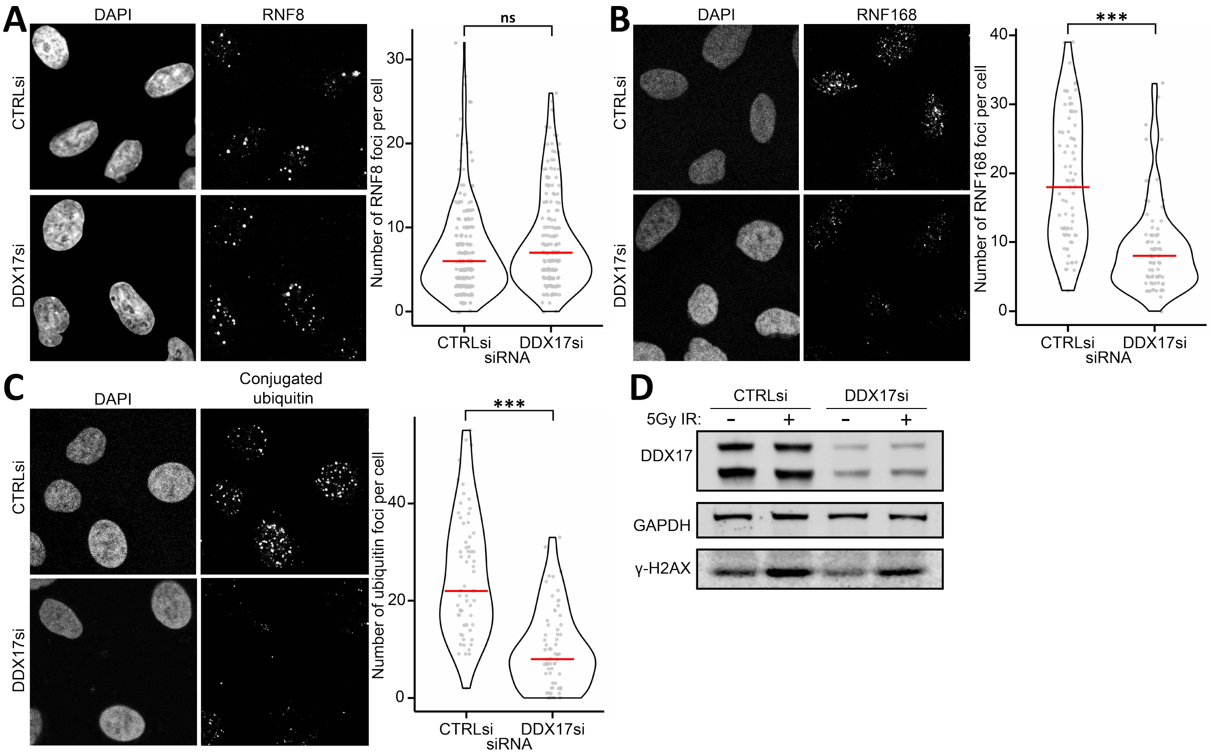

Supplemental Figure 4: DDX17 is required for RNF168 recruitment and histone ubiquitylation at DSBs. **(A)** Left: representative images of immunofluorescence of RNF8 in A549 cells treated with 5Gy IR with either control or DDX17 siRNA, right: RNF8 foci per cell quantification of a minimum of 198 cells across 3 biological replicates, red line is the median. Statistics were done using an unpaired directional Wilcoxon test not significant (ns). **(B)** Same as (A) but for RNF168 immunofluorescence in a minimum of 80 cells across 2 biological replicates, ***p<0.001. **(C)** Same as (A) but for conjugated ubiquitin immunofluorescence in a minimum of 68 cells across 2 biological replicates, ***p<0.001. **(D)** Western blot of DDX17 knockdown and 5Gy IR treatment in A549 cells.

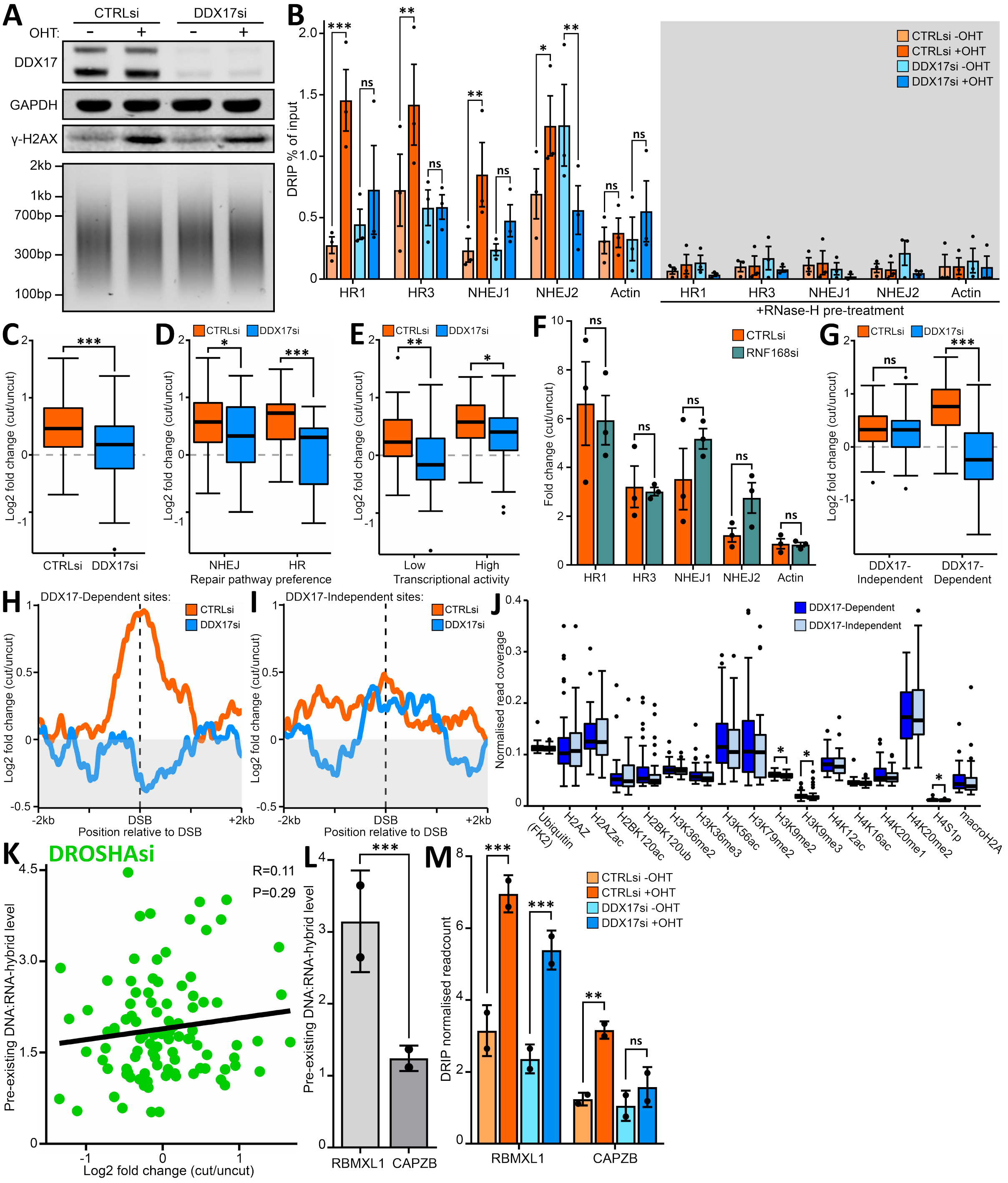

Supplemental Figure 5: DDX17 generates DNA:RNA-hybrids around DSBs at low DNA:RNA-hybrid loci. **(A)** Top is western blot validating DDX17 knockdown and 4 hour 300nM 4-hyroxytamoxifen treatment in DIvA cells, bottom is agarose gel validating DNA sonication fragmentation pattern for the DRIP. **(B)** DNA:RNA IP (DRIP) qPCR around HR and NHEJ repaired DNA break sites and an undamaged site in actin exon 5 in DIvA cells treated with 300nM 4-hydroxytamoxifen treatment for 4 hours and with either control or DDX17 siRNA. Bar represents the mean % of input for the IP of 3 biological replicates, error bar is SEM, points are individual replicate values. Right is the RNase-H pre-treated negative controls. Statistics

were done with a paired t-test, *p<0.05, **p<0.01, ***p<0.001. **(C)** Boxplot of DRIP-seq log2 fold change of damaged(+300nM hydroxytamoxifen for 4 hours))/ undamaged(-hydroxytamoxifen) DRIP read coverage at the ASISI induced DSB sites treated with either control or DDX17 siRNA. Statistics done with an unpaired, directional Wilcoxon test, ***p<0.001. **(D)** Same as (C) but DSB sites are split between those prone to either NHEJ or HR repair, *p<0.05. **(E)** Same as (C) but DSB sites are split between into high and low transcriptional activity groups, **p<0.01. **(F)** DRIP qPCR around HR and NHEJ repaired DNA break sites in the DIvA cell system and an undamaged site in actin exon 5. Bar represents the mean fold change of damaged (+OHT 4 hours) over undamaged (-OHT) for the IP % of input for 3 biological replicates, error bar is SEM. Statistics were done with a paired t-test, *p<0.05, **p<0.01, ***p<0.001. **(G)** Same as (C) but DSB sites are split into DDX17-dependent and DDX17-independent groups. **(H)** DRIP-seq DSB centred metagene analysis of DDX17-dependent loci cut by the AsiSI restriction endonuclease in the DIvA cell system, y-axis is log2 fold change of damaged/undamaged normalised readcounts. **(I)** Same as (G) but for DDX17-independent DSB loci. **(J)** ChIP-seq read coverage at DDX17-dependent and DDX17-independent DSB loci for various histone modifications. **(K)** Correlation plot of pre-existing DNA:RNA-hybrid level at 99 ASISI induced DSB loci against the Log2 fold change of damaged/undamaged DNA:RNA-hybrid level in DIvA cells treated with *DROSHA* siRNA, statistics were done with Pearson correlation testing. **(L)** Pre-existing DNA:RNA-hybrid level at the AsiSI induced DSB sites in the RBMXL1 and CAPZB genes. **(M)** DRIP read coverage at the AsiSI induced DSB sites in the high DNA:RNA-hybrid RBMXL1 locus and the low DNA:RNA-hybrid CAPZB locus in DIvA cells treated with or without 4-hydroxytamoxifen and with either control of DDX17 siRNA.

Supplemental Table 1: List of antibodies used for western blot (WB) or immunofluorescence (IF).

Antibody Target	Application	Supplier	Catalogue number	Dilution WB	Dilution IF
DDX17	WB	SCBT	sc-271112	1:1000	N/A
53BP1	WB, IF	Novus	NB100-305	1:1000	1:600
BRCA1 (D-9)	WB, IF	SCBT	sc-6954	1:500	1:100
Vinculin	WB	Abcam	Ab18058	1:5000	N/A
γ-H2AX	WB, IF	Merck	05-636	1:1000	1:600
RNF8	WB, IF	SCBT	sc-271462	1:500	1:50
RNF168	WB, IF	SCBT	ABE367	1:500	1:100
Conjugated Ubiquitin (FK2)	IF	Merck	04-263	N/A	1:200
GAPDH	WB	SCBT	sc-32233	1:10,000	N/A
DDX17	WB	Abcam	ab24601	1:1000	N/A
53BP1	WB, IF	Novus	NB100-304	1:2500	1:500
Tubulin (B512)	WB	Sigma	T5168	1:10,000	N/A
RAP80	IF	Bethyl	A300-763A-T	N/A	1:100
RPA32	IF	Abcam	Ab2175	N/A	1:250

Supplemental Table 2: List of primers used in the resection assay.

Target	Restriction enzyme	Primer sequences	Amplicon (hg38)
HR1 +200	BanI	FWD:ACCATGAACGTGTTCCGAAT REV:GAGCTCCGCAAAGTTTCAAG	chr22:38468231-38468461
HR1 +950	BanI	FWD:ACAGATCCAGAGCCACGAAA REV:CCCACTCTCAGCCTTCTCAG	chr22:38468951-38469111
HR2 +740	BanI	FWD:GTCCCCTCCCCCACTATTT REV:ACGCACCTGGTTTAGATTGG	chr20:32359128-32359340
HR2 +2000	BanI	FWD:GTTCCTGTTATGCGGGTGTT REV:TGGACCCCAAATTCCTAAAG	chr20:32360514-32360701
No DSB	HindIII-HF	FWD:ATTGGGTATCTGCGTCTAGTGAGG REV: GACTCAATTACATCCCTGCAGCT	chr22:22799150-22799291

Supplemental Table 3: List of primers used in DRIP-qPCR.

Target	Primer sequences	Amplicon (hg38)
HR1	FWD:CCGCCAGAAAGTTTCCTAGA REV:CTCACCCTTGCAGCACTTG	chr22:38468175-38468329
HR2	FWD:CCTAGCTGAGGTCGGTGCTA REV:GAAGAGTGAGGAGGGGGAGT	chr20:32358116-32358311
NHEJ1	FWD:ATCGGGCCAATCTCAGAGG REV:GCGACGCTAACGTTAAAGCA	chr6:89638541-89638692
NHEJ2	FWD:GGTGCCCACAGCTCTCTATG REV:GAAGCCAGAGGAGTGTCCTG	chr9:29212545-29212742
Actin	FWD:GTGACACAGCATCACTAAGG REV:ACAGCACCGTGTTGGCGT	chr17:81511013-81511146
